# Supplementary material for: Cohort profile: an observational longitudinal data collection of health aspects in a cohort of female cancer survivors with a history of pelvic radiotherapy—a population-based cohort in the western region of Sweden
Source: BMJ Open. 2021 Jul 20;11(7):e049479. doi: 10.1136/bmjopen-2021-049479 (PMC8296786; doi:10.1136/bmjopen-2021-049479)
Supplement: Author's manuscript [file bmjopen-2021-049479.draft_revisions.pdf]

BMJ Open is committed to open peer review. As part of this commitment we make the peer review history of every article we publish publicly available.

When an article is published we post the peer reviewers' comments and the authors' responses online. We also post the versions of the paper that were used during peer review. These are the versions that the peer review comments apply to.

The versions of the paper that follow are the versions that were submitted during the peer review process. They are not the versions of record or the final published versions. They should not be cited or distributed as the published version of this manuscript.

BMJ Open is an open access journal and the full, final, typeset and author-corrected version of record of the manuscript is available on our site with no access controls, subscription charges or pay-per-view fees (http://bmjopen.bmj.com).

If you have any questions on BMJ Open's open peer review process please email info.bmjopen@bmj.com

BMJ Open

Cohort profile: An observational longitudinal study examining health in a cohort of female cancer survivors with a history of pelvic radiotherapy, a population-based cohort in the Western Region of Sweden

Journal:	*BMJ Open*
Manuscript ID	bmjopen-2021-049479
Article Type:	Cohort profile
Date Submitted by the Author:	25-Jan-2021
Complete List of Authors:	Åkeflo, Linda; University of Gothenburg Sahlgrenska Academy, Department of Oncology<br>Dunberger, Gail; Ersta Sköndal Bräcke högskola - Campus Ersta, Department of health Care Sciences<br>Elmerstig, Eva; Malmö Universitet, Centre for Sexology and Sexuality studies<br>Skokic, Viktor; University of Gothenburg Sahlgrenska Academy, Department of Oncology<br>Steineck, Gunnar; University of Gothenburg Sahlgrenska Academy, Department of Oncology<br>Bergmark, Karin; University of Gothenburg Sahlgrenska Academy, Department of Oncology
Keywords:	Adult oncology < ONCOLOGY, Gastrointestinal tumours < ONCOLOGY, Gynaecological oncology < ONCOLOGY, Radiation oncology < RADIOTHERAPY

SCHOLARONE™
Manuscripts

I, the Submitting Author has the right to grant and does grant on behalf of all authors of the Work (as defined in the below author licence), an exclusive licence and/or a non-exclusive licence for contributions from authors who are: i) UK Crown employees; ii) where BMJ has agreed a CC-BY licence shall apply, and/or iii) in accordance with the terms applicable for US Federal Government officers or employees acting as part of their official duties; on a worldwide, perpetual, irrevocable, royalty-free basis to BMJ Publishing Group Ltd ("BMJ") its licensees and where the relevant Journal is co-owned by BMJ to the co-owners of the Journal, to publish the Work in this journal and any other BMJ products and to exploit all rights, as set out in our licence.

The Submitting Author accepts and understands that any supply made under these terms is made by BMJ to the Submitting Author unless you are acting as an employee on behalf of your employer or a postgraduate student of an affiliated institution which is paying any applicable article publishing charge ("APC") for Open Access articles. Where the Submitting Author wishes to make the Work available on an Open Access basis (and intends to pay the relevant APC), the terms of reuse of such Open Access shall be governed by a Creative Commons licence – details of these licences and which Creative Commons licence will apply to this Work are set out in our licence referred to above.

Other than as permitted in any relevant BMJ Author's Self Archiving Policies, I confirm this Work has not been accepted for publication elsewhere, is not being considered for publication elsewhere and does not duplicate material already published. I confirm all authors consent to publication of this Work and authorise the granting of this licence.

For peer review only

BMJ Cohort profile

Cohort profile: An observational longitudinal study examining health in a cohort of female cancer survivors with a history of pelvic radiotherapy, a population-based cohort in the Western Region of Sweden

Linda Åkeflo[a]*, Gail Dunberger[b] ,Eva Elmerstig[c], Viktor Skokic[a], Gunnar Steineck[a] , Karin Bergmark[a]

[a]Department of Clinical Cancer Epidemiology, Department of Oncology, Institute of Clinical Science, The Sahlgrenska Academy, University of Gothenburg, Gothenburg, Sweden; [b]Department of Health Care Sciences, Ersta Sköndal Bräcke University College, Stockholm, Sweden; [c]Centre for Sexology and Sexuality studies, Malmö University, Malmö, Sweden,

*Correspondence: L. Åkeflo, Division of clinical Cancer Epidemiology, Department of Oncology, Sahlgrenska Academy at University of Gothenburg, 413 45 Gothenburg, Sweden. Tel: +46 732 017972. Fax: +46 31 823931.

E-mail: linda.akeflo@vgregion.se

For peer review only

BMJ Cohort profile

ABSTRACT

Purpose: In response to the need for more advanced and longitudinal data concerning long-term radiotherapy-induced late effects and states among female cancer survivors, the study “Health among women after pelvic radiotherapy”, was established.

Participants: Since 2011 and ongoing, participants are recruited from a population-based study-cohort including all female cancer patients over 18 years old, treated with pelvic radiotherapy with curative intent at Sahlgrenska University Hospital in Gothenburg, Sweden in the Western Region, covering 1.7 million of the Swedish population. The dataset here accounted for baseline data provided by 605 female cancer survivors and three-month follow-up data from 260 individuals with gynecological-, rectal- and anal cancer collected during a six-year period.

Findings to date: The data collected include self-reports of demographics, physical symptoms, and cancer-treatment details. The cohort is currently used for research projects in radiation-induced intestinal syndromes and late adverse effects affecting sexuality, urinary tract, lymphatic system, and physical activity. These projects include evaluation of interventions developed and provided in a nurse-led clinic. A recently published study report cancer survivors need support for additional rehabilitation when having fecal- and urinary-incontinence to increase physical activity levels.

Future plans: This cohort will be expanded to comprise the entire data collection from 2011-2020, including baseline data, data from the three months-, and the one-year follow-up data after interventions. The data will be used to study conditions and treatment-induced late-effects pre-, and post-interventions. The data collection and the cancer rehabilitation-model outlined in this paper have potential to contribute to the future development of treatment for radiotherapy-induced late effects among pelvic cancer survivors.

Keywords: *Female cancer survivors, Treatment-induced late-effects, Pelvic radiotherapy, Cancer rehabilitation, Nurse-led clinic*

Strengths and limitations

- The large dataset consists of a population-based cohort, which creates good conditions for studying cancer survivors without selection-induced problems.
- Results of this study will provide new information on a wide range of treatment-induced cancer survivorship diseases and states. The interventions outlined in this paper have the potential to contribute to understand manifestations of treatment-induced cancer survivorship diseases and states, and to the future development of evidence-based management strategies in pelvic cancer rehabilitation.
- Non-participation and loss to follow-up may affect the results.
- There is no information from study-participants´ reasons for drop-off.
- Our cohort includes data collected in Sweden, which may limit the generalizability to other populations.

For peer review only - http://bmjopen.bmj.com/site/about/guidelines.xhtml

Introduction

An increasing number of individuals live longer after a cancer diagnosis than was common in the past, and more and more patients are now living with treatment-induced late-effects, treatment-induced cancer survivorship diseases, and survivorship states [1-3]. Pelvic radiotherapy affects the intestinal and urinary tract, the lymphatic system, and sexual health [4-6]. In this video at https://www.jostrust.org.uk/video/letstalk-pelvic-radiation-disease-after-cervical-cancer, some of more than 20 million cancer survivors in Europe express how radiation-induced impaired intestinal health affects them as cancer survivors. In Sweden, approximately 3500 women are diagnosed with pelvic cancer (gynecological-, anal-, and rectal cancer) every year [7]. In 2011, we started a nurse-led clinic focusing on treatment-induced late effects among pelvic cancer survivors with the intent to develop self-care strategies and treatments. We have previously reported on the benefits of the clinic regarding the resulting improved quality of life and psychosocial wellbeing among female pelvic-cancer survivors [8].

In this paper, we describe a study cohort in a continuous data collection procedure, and interventions performed at a nurse-led clinic that have been ongoing since 2011. We collect data from female cancer survivors with a history of pelvic radiotherapy in western Sweden, an area that includes 20 percent of the Swedish population. The Swedish register-tradition offers good conditions for studying cancer survivors without selection-induced problems. The dataset contains information on needs pre-intervention and post-intervention from women that have received pelvic radiotherapy at Sahlgrenska University Hospital 2007-2016. Female cancer survivors referred to the nurse-led clinic are also invited to participate in the study. This paper outlines the systematic data-collection and the characteristics of the study participants, data and information that can be employed in future analyses to then be reported.

For peer review only

Cohort description

Setting

In 2009 the Swedish government proposed a new regulation: “A National Cancer Strategy” (SOU2009:1) [9]. In collaboration with county councils and regions, six Regional Cancer Centers (RCC) were established. Based on the national strategy proposals, the RCC in western Sweden finances a nurse-led clinic at Sahlgrenska University Hospital. The nurse-led clinic was founded in 2011 by a physician senior consultant with a PhD degree who has specialized in gynecological oncology, together with an oncology nurse with a PhD degree in

For peer review only - http://bmjopen.bmj.com/site/about/guidelines.xhtml

oncology. Currently, the team consists of three clinical oncology nurses specially trained in understanding and working on issues concerning cancer survivorship. The cancer survivors receive education about radiotherapy-induced late-effects, basic anatomy and physiology and together with the nurse decide on actions for supportive care regarding medication, nutrition, and coping with sexual, psychological and social challenges. In case of specific needs, intercurrent diseases or suspected recurrence of cancer, the patient meets the medical senior consultant who has the primary medical responsibility for the clinic. The medical senior consultant meets the team regularly and discusses planning for medication with the staff member who will issue the prescriptions. The health-care program developed in the clinic is based on programs developed by others and on our previous studies in the area [4,10-16].

Participants

Study participants are recruited from two different cohorts; 1) a population-based study-cohort group including all female cancer patients treated with curative intent from 2007 and onwards continuously identified from medical records at Sahlgrenska University Hospital in Sweden and, 2) a referred patient group including all female patients referred to the rehabilitation clinic. Inclusion criteria are female cancer survivors with a history of pelvic radiotherapy, at least six months since completed radiotherapy. Exclusion criteria are: Recurrence of cancer, and women physically and cognitively unable to understand and answer the questionnaire or who do not understand Swedish at all.

Invited women

An introductory letter is sent to eligible study participants. Shortly after, a research secretary phones and gives them oral information, asks them if they are willing to participate in the study and to give permission to be sent a written informed consent and the questionnaire. When the baseline questionnaire is returned, the participant receives an invitation to come to the nurse-led clinic where individual healthcare interventions are performed. The code number from the questionnaire (followed throughout the data collection) is entered into the patient-database FileMaker Pro17 Advanced®, specifically designed to suit the study. Three and twelve months after the completed intervention, the study participant receives a follow-up questionnaire. In case questionnaires are delayed or missing, the research secretary who keeps track of each patient sends two reminders at time-points determined in advance, see figure 1.

For peer review only - http://bmjopen.bmj.com/site/about/guidelines.xhtml

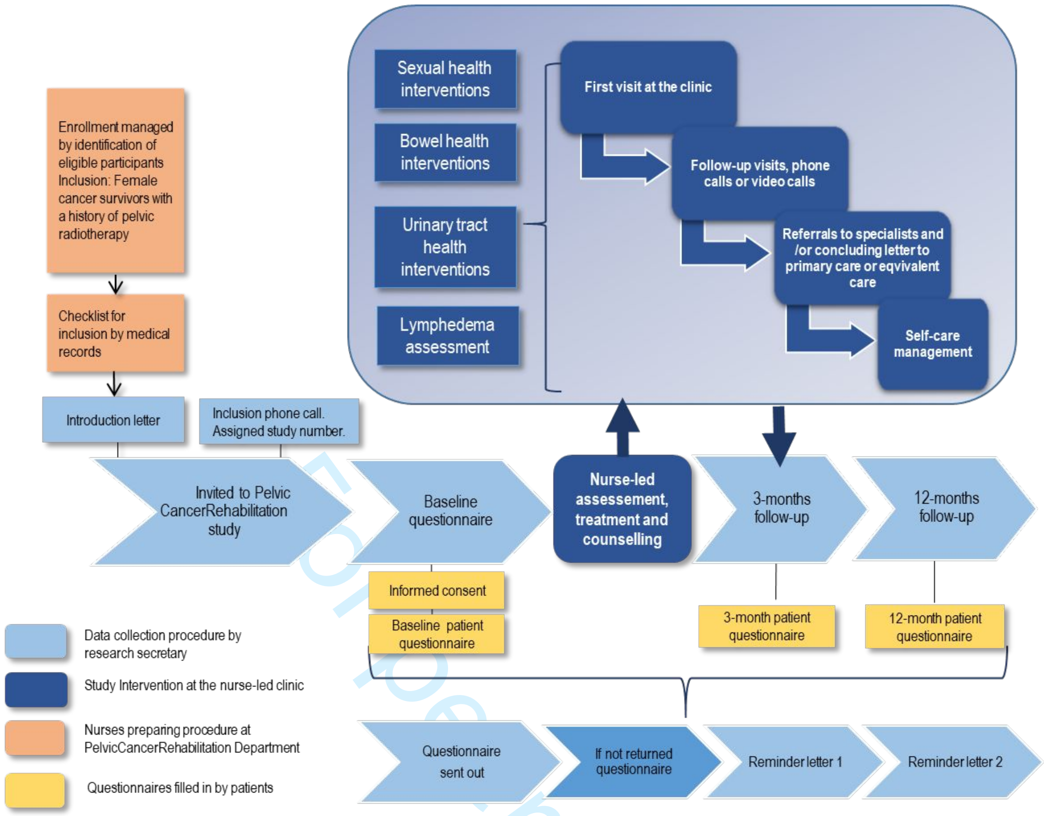

Figure 1. *Flowchart of Pelvic cancer rehabilitation ongoing data collection procedure and the procedure for patient inclusion, contact with participants and the follow up points for questionnaires.*

Referred patients

Patients referred from oncology health care providers, general practitioners and private referrals that meet inclusion criteria, are also invited to participate in the study. The data collection procedure followed the same principles as followed for the group of invited women.

Study-specific questionnaires

As shown in table 1, the baseline questionnaire consists of 175 questions, divided into eight main sections and one concluding chapter. Information about the frequency, intensity, duration, and quality of each symptom and the degree of the distress it causes is obtained. One question is cited here as an example: "How many times (approximately) have you had bowel movements per week, during the last six months?" with possible answers "About every other day", "Fewer than once a day", "Once a day", "Up to twice a day", "2-3 times per day", "3-4 times per day", "5 times or more, per day". To facilitate statistical analyses, the answers are coded into values, ranked and placed into groups. Variables such as age and number of children are categorized into groups.

For peer review only - http://bmjopen.bmj.com/site/about/guidelines.xhtml

Table 1. The study-specific baseline questionnaire, divided into eight sections and one concluding section

Section	Question areas
Sociodemographic	Gender<br>Age<br>Marital status<br>Children<br>Level of education<br>Employment status
Quality of life and wellbeing	Quality of life<br>Depression<br>Worrying<br>Anxiety
Body-perception and self-image	Femininity<br>Self-esteem<br>Fertility<br>Childbirth<br>Vaginal and perianal injury related to childbirth or physical trauma
Intestinal and defecation habits	Loose stool<br>Fecal incontinence<br>Urgency to defecation<br>Excessive gas<br>Abdominal pain<br>Having stoma<br>Medical treatments related to intestinal symptoms
Micturition habits and urinary-tract symptoms	Urinary frequency<br>Urgency to urinate<br>Nocturia<br>Having a urine catheter<br>Medical treatment due to symptoms
Sexual health	Menopause<br>Use of systemic hormone replacement therapy<br>Use of topical estrogen<br>Impaired lubrication<br>Vaginal shortness<br>Vaginal inelasticity<br>Dyspareunia
Sexual abuse	Experienced sexual abuse<br>Experience sexual harassment<br>Age at sexual abuse or harassment<br>To which extend the exposure affects sexual life
Lymphedema	Heaviness in legs, genitals and abdomen<br>If diagnosed with lymphedema, current lymphedema treatment
Concluding chapter	Self-reported needs<br>An offer to visit the nurse-led clinic for assessment and counselling

Several sections allow free comments

For peer review only

Additionally, the questionnaire addresses the extent to which the symptoms affect social functioning. In the concluding section, participants are invited to visit the nurse-led clinic. Some questions serve to rank the patients' most distressing symptoms. In the follow-up questionnaires the study-participants’ health-status and symptoms are measured, and interventions performed at the nurse-led clinic are evaluated.

For peer review only - http://bmjopen.bmj.com/site/about/guidelines.xhtml

Diagnostic and alleviative means

At the first visit to the clinic, the questionnaire serves as a foundation for the interventions. The patient´s current health status reveals the needs that are in focus. Current symptoms and self-care strategies are discussed, and new strategies suggested and decided on together with the patient. The duration of the contact and the number of follow-ups varies from one month to one year, depending on the symptoms and the effect of treatments and interventions. Cerna et al (17) observed in a study with an educational perspective that patients and nurses in current settings can together create tailor-made solutions. The nurses focus on encouraging the patients' self-reflection and keep up the patient's motivation to continue to engage in self-care.

Intestinal health

The assessment is based on five syndromes: urgency to defecate, fecal leakage, excessive gas production, excessive mucus discharge and blood discharge [17]. Symptoms such as leakage of mucus and blood, abdominal bloating, signs of bacterial overgrowth and bile-salt malabsorption are also assessed. Diagnostic means including blood test, for example, routine blood screening, electrolytes, Vitamin B12 and serum Mg2 are taken when indicated. An algorithm developed by the research group serves as a guide in the clinical setting [8]. A mobile application has been developed that was specially designed for radiation-induced intestinal syndromes and can show graphs of variation in intestinal health over time. The application serves as a complement to existing methods for self-assessment and patients' self-management and helps to support the nurse- and patient's conversation and decision-making [18,19]. The objective of the interventions is to restore or improve intestinal health and includes medical treatment, pelvic-floor muscle training and techniques of cognitive training to control the intestinal function. Details of these interventions can be accessed elsewhere [8,20].

Sexual health

The PLISSIT-model (Permission, Limited Information, Specific Suggestions, and Intensive Therapy) developed by Annon [21] is used for addressing sexual-health concerns. The first three levels in the model are helpful for most patients. When specific suggestions are not sufficient, the patient is recommended to take part in Intensive Therapy, sometimes given in the current setting, at other times with a person named in a referral to a sexologist or psychotherapist. Female sexual dysfunction is a complex condition including diverse aspects, definitions, and classifications [22]. To assess and treat sexual dysfunction, previous sexual practices and experiences are discussed with attention given to patients' integrity, special needs, and preferences. Structural information about common physical late effects regarding vaginal changes and sexual problems is provided, as well as information

For peer review only - http://bmjopen.bmj.com/site/about/guidelines.xhtml

on psychological issues influencing sexual health, in accordance with evidence-based knowledge and practice in the field [4,13,23-25,1]. Patients receive information about and instructions on use of vaginal dilator therapy and topical estrogen, and they are also given guidance and suggestions regarding a lack of desire. When requested, specific sexual devices, films, and literature are suggested to encourage and help the patient to regain sexual function as well as to regain body, self-esteem, and intimacy.

Sexual abuse

Cancer patients with a history of sexual abuse have an increased risk of sexual problems after cancer treatment and a large proportion of women with cervical cancer and dyspareunia post-cancer-treatment have been sexually abused [26]. In reported cases, experiences of sexual abuse are carefully discussed with the patient and in some cases, this leads to referral to psychologists or therapists.

Urinary-tract health

Symptoms of urgency, nocturia, urinary retention, urinary-tract infection, and pelvic pain are addressed. Diagnostic means includes evaluation of urinary frequency and of symptoms divided into irritative symptoms, obstructive symptoms, and bleeding. A 72-hour voiding diary including urine volume and fluid intake is a helpful resource for this discussion. Consulting and guidance are given by making recommendations about topical estrogen, pelvic floor muscle training, medical treatment, self-care modifications and behavioral interventions, in accordance with previous evidence-based knowledge [27,28]. In case of severe urinary-tract symptoms, referrals are sent to urotherapists, physiotherapists or urologists.

Lymphedema

Lower limb lymphedema is a common non-curable chronic complication with multi-factorial pathophysiology [29]. Early detection and treatment of lymphedema is essential in order to prevent complications. Self-reported or objectively assessed swelling or heaviness in the lower limbs leads to referral to a lymph-therapist. The primary therapy is the use of individually tailored compression garments and second-line therapy consists of manual lymphatic drainage with for example intermittent use of pumps and other self-care strategies [30,31].

Statistical analyses

Initially, data from the questionnaires were entered into EpiDataSoftware® version 3.1 (EpiData Association). Since only one answer is allowed for each question, the procedure was to enter the first choice every other time and the second choice the other time in case a question was marked with two answers. In order to make analysis

For peer review only

of open-ended questions possible, the answers to these were transcribed in Microsoft Word (2016). R version 3.5.2. was used for statistical analysis of the data. The results will be reported in means, medians, and percentages.

Ethics

All procedures performed in studies involving human participants were in accordance with the ethical standards of the regional ethics review board in Gothenburg (D 686-10) and with the 1964 Helsinki declaration and its later amendments. The research was approved by the Regional Ethics Review Board in Gothenburg (D 686-10), Gothenburg University. Written informed consent was obtained from all individual participants included in the study.

Findings to date

From January 2011 to June 2017, we identified a total of 791 patients in the population-based cohort that met the eligibility criteria. Of the total sample, 684 (86%) individuals gave oral consent to participate in the study and of these 464 (68%) completed the baseline questionnaire.

During the same period, 184 referred patients met the inclusion criteria and were invited to participate in the study. One-hundred-and-forty-one (76.6%) participants completed the baseline questionnaire and of these 131 (92 %) participants took part in interventions.

For peer review only - http://bmjopen.bmj.com/site/about/guidelines.xhtml

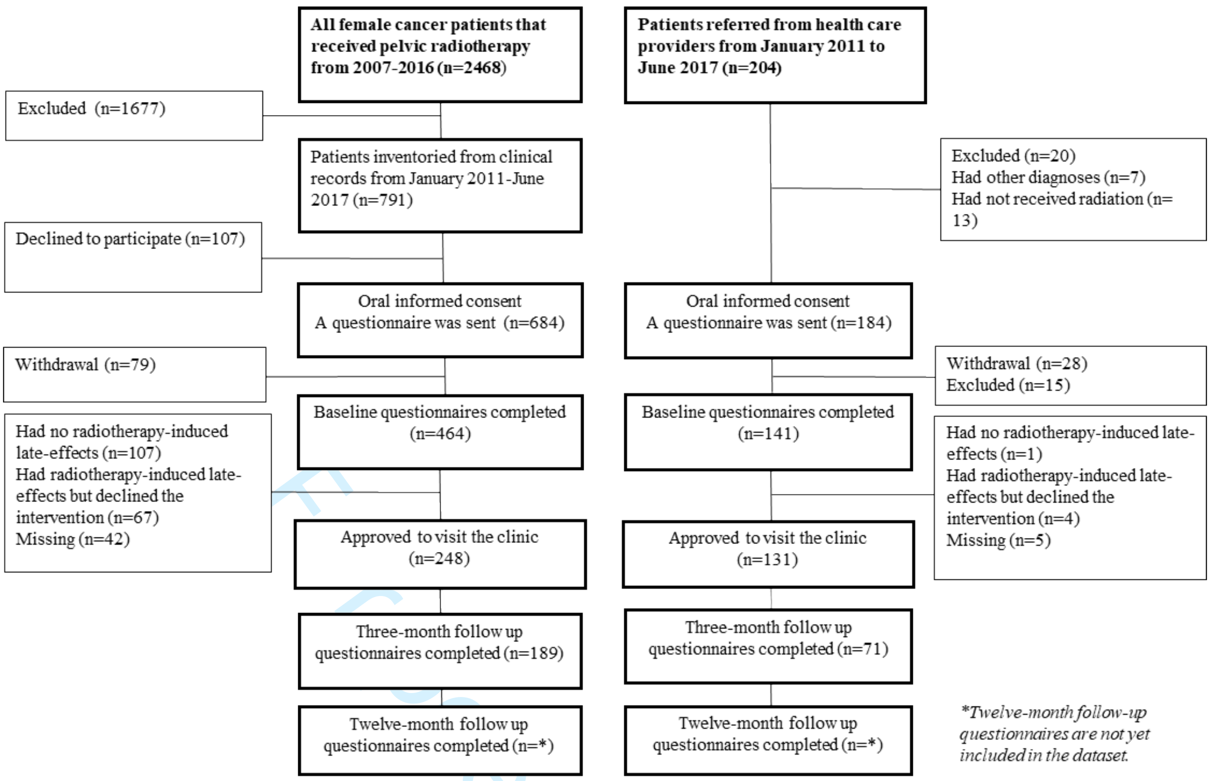

Figure 2. *Schematic diagram of data collected from two different study-cohorts from January 2011 to June 2017; a population-based cohort and patients referred from oncology health care providers, general practitioners and private referrals that met the inclusion criteria. The diagram includes study response rate, completeness of questionnaires and reasons for loss of participants.*

The responses and completeness of the questionnaires from both study groups are shown in the schematic diagram, see figure 2. In table 2, we present the baseline characteristics and demographics in the dataset that consists of 605 participants: 464 (76.7 %) from the invited cohort and 161 (23.3 %) from the referred cohort.

For peer review only

Table 2. Characteristics of the study participants

Variable	Total	Invited	Referral
**Participants, n (%)**	605	464 (76.7)	141 (23.3)
**Cancer type, n (%)**			
Endometrial cancer	216 (35.7)	181 (39.0)	35 (24.8)
Cervical cancer	132 (21.8)	80 (17.2)	52 (36.9)
Ovarian cancer	2 (0.3)	1 (0.2)	1 (0.7)
Vaginal cancer	5 (0.8)	3 (0.6)	2 (1.4)
Vulvar cancer	21 (3.5)	19 (4.1)	2 (1.4)
Anal cancer	80 (13.2)	58 (12.5)	22 (15.6)
Rectal cancer	145 (24.0)	122 (26.3)	23 (16.3)
Other	4 (0.7)		4 (2.8)
**Age in years**			
Mean	64.5	66.5	57.6
SD	12.6	11.5	13.6
Missing, (%)	11	7 (1.5)	4 (2.8)
**Years since radiotherapy, grouped**			
0	35 (5.8)	6 (1.3)	29 (20.6)
1	219 (36.1)	166 (35.8)	53 (37.6)
2	98 (16.1)	86 (18.5)	12 (8.5)
3	139 (23.0)	131 (28.2)	8 (5.7)
≥4	105 (17.3)	69 (14.9)	36 (25.5)
Missing	9 (1.5)	6 (1.3)	3 (2.1)
Mean	2.6	2.2	3.9
SD	3.4	1.2	6.7
**Cancer treatment, n (%)**			
External radiotherapy, only	145 (24.0)	101 (21.8)	44 (31.2)
External radiotherapy and brachytherapy	20 (3.3)	16 (3.4)	4 (2.8)
External radiotherapy, brachytherapy and surgery	180 (29.7)	157 (33.8)	23 (16.3)
External radiotherapy and surgery	260 (43.0)	190 (40.9)	70 (49.6)
**Marital status, n (%)**			
Married or living with a partner	402 (66.4)	309 (66.6)	93 (66.0)
Widow	70 (11.6)	59 (12.7)	11 (7.8)
Has a partner but lives alone	30 (5.0)	15 (3.2)	15 (10.6)
Single	102 (17.0)	80 (17.2)	22 (15.6)
Missing	1 (0.2)	1 (0.2)	
**Education level, n (%)**			
Elementary school	173 (29.1)	150 (32.3)	23 (16.3)
Secondary school	227 (38.2)	169 (36.4)	58 (41.1)
Collage/University	194 (32.0)	135 (32.3)	59 (41.8)
Missing	11 (1.8)	10 (2.2)	1 (0.7)
**Employment status, n (%)**			
Student	4 (0.7)	3 (0.6)	1 (0.7)
Unemployed job seeker	12 (2.0)	10 (2.2)	2 (1.4)
Employed	162 (27.0)	116 (25.0)	46 (32.6)
Housewife	4 (0.7)	2 (0.2)	2 (1.4)
On sick leave	54 (9.0)	17 (3.7)	37 (26.2)
Disability pension	35 (5.7)	26 (5.6)	9 (6.4)
Retired	328 (54.7)	284 (61.2)	44 (31.2)
Missing	6 (1.0)	6 (1.3)	
**Resident, n (%)**			
In a big city	182 (30.6)	131 (28.2)	51 (36.2)
Small or medium sized city	309 (50.2)	244 (52.6)	61 (43.3)
On the countryside	116 (18.9)	87 (18.8)	29 (20.6)
Missing	2 (0.3)	2 (0.4)	
**Smoking, n (%)**			
Not smoking	448 (74.0)	339 (73.1)	109 (77.3)
Smoking	67 (11.0)	52 (11.2)	17 (12.1)
Missing	88 (14.5	73 (15.7)	15 (10.6)

N (number) and proportion (%) of women is presented. SD = Standard Deviation

For peer review only

The majority of the study participants had a history of gynecological cancer and had been treated with radiotherapy in combination with surgery. In the population-based study-cohort, the mean age was higher and twice as many were retired compared to the referred group. In the referred group, twice as many were on sick leave. In total, 379 (63 %) of 605 study participants were approved to visit the clinic, see table 3. Sixty-seven (14.4 %) of the women in the population-based study-group that declined to visit the clinic report that they had radiotherapy-induced late-effects.

Table 3. Number (N) and proportion (%) of study-participants that approved to visit the clinic

	Total N (%)	Invited N (%)	Referred N (%)
No, I have no late effects and do not need to visit the clinic	108 (17.8)	107 (23.1)	1 (0.7)
No, I have late effects, but I do not want to visit the clinic	71 (11.7)	67 (14.4)	4 (2.8)
Yes, I want to visit the clinic	379 (62.6)	248 (53.4)	131 (92.9)
Missing	47 (7.8)	42 (9.1)	5 (3.5)

For peer review only

Strengths and limitations

The data collection has generated a large dataset consisting of patient entries from six months to several years post pelvic radiotherapy treatment. Over a period of six and a half years, almost 1000 female pelvic cancer survivors were invited to participate in the study, and this dataset consists of 605 cancer survivors. In the population-based cohort-group, 68 % completed a baseline questionnaire. Treatment and interventions were offered from physical, psychosocial and sexual perspective to patients from both the population-based cohort and the referred cohort and were evaluated. As shown in table 3, 53.4 % of study participants in the study population cohort were approved to visit the clinic, which may indicate the proportion of cancer survivors' unmet needs. At present, improved self-care strategies, increased clinical knowledge, and development of technology enable the possibilities for helping cancer-survivors manage and treat late effects. By manifesting treatment-induced cancer survivorship diseases and states, we believe that it may be more likely that effective treatments can be developed.

Cancer survivorship issues have advanced from being neglected to gradually being given increasingly greater attention in healthcare practice as well as in research. That is reflected by the development of national

For peer review only - http://bmjopen.bmj.com/site/about/guidelines.xhtml

guidelines, scientific conferences and national and international meetings in the area. Efforts are made to both understand the manifestation of treatment-induced late effects, as well as to find strategies to deal with late effects in pelvic cancer survivors. Twenty years have passed since researchers within our team [4] first observed that gynecological cancer patients suffer from vaginal changes affecting their sexual health. More recently, we have identified five syndromes impairing pelvic cancer survivors' intestinal health. The results published in 2017 [17] simplify the search for prevention, management, and for help in minimizing the occurrence and intensity of survivorship diseases. One promising ongoing study conducted by Schofield et al in Australia, evaluates a care program similar to ours [32,33]. Furthermore, in 2015 Andreyev et al [11] published a guide for management of intestinal problems, including an algorithm that in our opinion is a useful tool for clinicians to use. However, the pathophysiological changes, described in numerous previous studies are not yet fully understood [15-17]. Inflammatory and fibrotic processes in the gut wall are probably of importance in explaining the varying symptoms. The processes may relate to both the intestinal tract and other organs located in the pelvis. Hofsjö et al in our research group [34], recently studied vaginal changes and found morphological explanations for changes in the vaginal wall. Biopsies from the vaginal connective tissue affected by radiotherapy showed dense collagen and entangled elastin fibers, a finding that may explain common symptoms such as reduced vaginal elasticity during intercourse, reduced lubrication and dyspareunia.

In Sweden, promising steps are being taken towards developing a program of national coordination of cancer rehabilitation practice. The ongoing establishment of oncology nurse navigators [35] is also an initiative that has the potential to increase the supportive care to cancer survivors. In the 1980s, research in the psychological field [36] showed that patients have a high risk of anxiety related to oncology treatment. Recent studies report that patients have a lower risk of future anxiety and depression when addressing needs during treatment [37,38]. In our opinion, when planning future follow-up in clinical cancer care, these findings need to be taken into account, irrespective of the patients' cancer diagnosis. We suggest that healthcare in the future should provide advanced specialist expertise to handle severe treatment-induced late-effects.

Sexual concerns are generally not addressed or discussed as much as the patients would like [39], and patients generally wait for healthcare providers to bring up the topic [40]. In the current setting, sexual health conversations are integrated in clinical work, routines for assessment and treatment are created, and it becomes

For peer review only - http://bmjopen.bmj.com/site/about/guidelines.xhtml

clearer when a patient should be referred to specialists and sexologists. The PLISSIT-model [21] has been used for addressing sexual health concerns in our as well as in other different clinical settings. Clinical experiences show that sexual functioning might improve and even return to pre-diagnosis level through frequent follow-up, observations that are consistent with results from previous studies [41]. Considering parts of WHO´s definition; "sexual health requires a positive and respectful approach to sexuality and sexual relationships" [42], health care providers need to be able to talk openly about sexuality. To overcome common communication barriers, it has been suggested that health-care professionals should actively engage in training to improve their communication skills [43,44]. Our clinical experience reveals that several patients were relieved to be able to speak openly about sexual issues as well as about other private health issues. The baseline questionnaire seems to serve as a therapeutic tool that can be followed up in counseling.

In this study, we found it necessary for ethical reasons to offer all patients in the population the best available intervention. Hence, the observational study design was considered to be the best suited, a design previously shown to produce reasonably useful results [45]. We use self-reported data that are known to provide a wider range of responses than data collected using other data collection instruments [46]. To avoid information-related problems, we took preventive actions in advance by using questionnaires based on the clinometric method introduced, developed, used and described by us in previous research projects [4,6,47,5]. Furthermore, we employed epidemiological methods, as introduced into the survivor-field by a hierarchical step-model, to handle bias and confounding [48]. We consider that the wide range of time since completed treatment may be a weakness in the study but will probably also facilitate measurement of symptom progression in future analysis. The minor changes in the interventions made during the study carried out due to increased knowledge need to be considered in the analysis. Furthermore, the non-participation and loss to follow-up may affect the results. We can only speculate on the causes but possible reasons for the drop-offs may be lack of time, not being motivated or being unwilling to recall the experience of previously had cancer. Possible reasons for declining an offer to visit the clinic despite troublesome symptoms could be having too severe symptoms to travel to the clinic. Age-related problems and long-distance transportation can be other causes. The large number of population-based study participants is considered to be one of the strengths in this study since this creates good conditions for studying cancer survivors without selection-induced problems. The data collected from the referred patients will allow analysis of the prevalence of symptoms and unmet needs observed by other health-care providers. Since

For peer review only

For peer review only - http://bmjopen.bmj.com/site/about/guidelines.xhtml

the data were collected in Sweden, we do not know to what extent our future analysis will be applicable to other populations.

Our and others' understanding of the complexity and pathophysiological mechanisms of symptoms and late effects has increased during the study. Through recurrent lectures for patients and professionals, and by implementation of treatment strategies directly to patients, we return the acquired knowledge to the clinic and to the cancer survivors themselves. Worth mentioning is that current rehabilitation clinic serves as a model for similar clinics in other regions of Sweden, which is part of the national strategy financed by the Swedish government. The current nurse-led clinic is possibly the beginning of a future tertiary center to develop interventions and treatments to cancer survivors.

We believe that the data collection outlined in this paper and the interventions that continually evaluates will contribute to understand manifestations of treatment-induced cancer survivorship diseases and states, and to future development of evidence-based management strategies in pelvic cancer rehabilitation. Further results from the interventions will be reported in future papers.

Collaboration

Requests for specific research projects and collaborative work are encouraged and can be addressed to the corresponding author.

Patient and public involvement

Results from future studies from the female cancer survivor population-based cohort will be submitted for publication in peer-reviewed journals and presented at relevant conferences.

Acknowledgements

For peer review only

For peer review only - http://bmjopen.bmj.com/site/about/guidelines.xhtml

We thank all the women who are participating in this study, the oncology nurses Lisen Heden and Karin Gustafsson, and the assistant Elisabeth Bystedt at BäckencancerRehabiliteringen, Sahlgrenska University Hospital for their skilful work with study inclusion and study interventions. We also thank medical student Amanda Arnell for assistance with preparing the dataset and the Regional Cancer Center of Västra Götaland and Västra Götaland County Council for financially supporting BäckencancerRehabiliteringen at Sahlgrenska University Hospital, Gothenburg, Sweden.

Footnotes

Author's contributions:

1. Guarantor of integrity of the entire study: Gunnar Steineck
2. Study concepts and design: Karin Bergmark, Gunnar Steineck, Gail Dunberger
3. Data analysis: Linda Akeflo, Gail Dunberger, Karin Bergmark, Eva Elmerstig, Gunnar Steineck
4. Statistical analysis: Viktor Skokic
5. Manuscript preparation: Linda Akeflo, Karin Bergmark, Gail Dunberger, Eva Elmerstig
6. Manuscript editing: Linda Akeflo

All authors have read and approved the final manuscript.

Funding

This research received no specific grant from any funding agency in the public, commercial or not-for-profit sectors

Competing interests

The authors declare that they have no conflict of interest. The authors have full control of all primary data and agree to allow the journal to review the data if requested.

Patient and public involvement:

Patients and/or the public were not involved in the design, or conduct, or reporting, or dissemination plans of this research.

For peer review only

Patient consent for publication:

Not applicable.

Provenance and peer review:

Not commissioned; externally peer reviewed.

Data availability statement:

Data are available on reasonable request.

Abbreviations

N	Number
PLISSIT	Permission, Limited Information, Specific Suggestions and Intensive Therapy
RCC	Regional Cancer Center
SD	Standard Deviation
SOU	Statens Offentliga Utredningar (Swedish: National Public Inquiries)
WHO	World Health Organization

References

1. Allemani C, Matsuda T, Di Carlo V, Harewood R, Matz M, Niksic M, Bonaventure A, Valkov M, Johnson CJ, Esteve J, Ogunbiyi OJ, Azevedo ESG, Chen WQ, Eser S, Engholm G, Stiller CA, Monnereau A, Woods RR, Visser O, Lim GH, Aitken J, Weir HK, Coleman MP (2018) Global surveillance of trends in cancer survival 2000-14 (CONCORD-3): analysis of individual records for 37 513 025 patients diagnosed with one of 18 cancers from 322 population-based registries in 71 countries. Lancet (London, England) 391 (10125):1023-1075. doi:10.1016/s0140-6736(17)33326-3

2. Miller KD, Siegel RL, Lin CC, Mariotto AB, Kramer JL, Rowland JH, Stein KD, Alteri R, Jemal A (2016) Cancer treatment and survivorship statistics, 2016. 66 (4):271-289. doi:10.3322/caac.21349

For peer review only

For peer review only - http://bmjopen.bmj.com/site/about/guidelines.xhtml

3. Steineck G, Schmidt H, Alevronta E, Sjoberg F, Bull CM, Vordermark D (2016) Toward Restored Bowel Health in Rectal Cancer Survivors. Semin Radiat Oncol 26 (3):236-250. doi:10.1016/j.semradonc.2016.03.002

4. Bergmark K, Åvall-Lundqvist E, Dickman PW, Henningsohn L, Steineck G (1999) Vaginal Changes and Sexuality in Women with a History of Cervical Cancer. New England Journal of Medicine 340 (18):1383-1389. doi:doi:10.1056/NEJM199905063401802

5. Dunberger G, Lind H, Steineck G, Waldenstrom AC, Nyberg T, Al-Abany M, Nyberg U, Vall-Lundqvist E (2010) Self-reported symptoms of faecal incontinence among long-term gynaecological cancer survivors and population-based controls. Eur J Cancer 46 (3):606-615. doi:10.1016/j.ejca.2009.10.023

6. Lind H, Waldenstrom AC, Dunberger G, al-Abany M, Alevronta E, Johansson KA, Olsson C, Nyberg T, Wilderang U, Steineck G, Avall-Lundqvist E (2011) Late symptoms in long-term gynaecological cancer survivors after radiation therapy: a population-based cohort study. British journal of cancer 105 (6):737-745. doi:10.1038/bjc.2011.315

7. Socialstyrelsen (2017) Statistical Database. https://www.socialstyrelsen.se/en/statistics-and-data/statistics/.

8. Dunberger G, Bergmark K (2012) Nurse-led care for the management of side effects of pelvic radiotherapy: what does it achieve? Curr Opin Support Palliat Care 6 (1):60-68. doi:10.1097/SPC.0b013e32834f6a95

9. 2009:11 S (2009) A National Strategy for the Future - Summary. doi:https://www.regeringen.se/49b6a7/contentassets/e343b40615eb46b395e5c65ca38d1337/summary-sou-200911

10. Andreyev J (2007) Gastrointestinal symptoms after pelvic radiotherapy: a new understanding to improve management of symptomatic patients. Lancet Oncol 8 (11):1007-1017. doi:10.1016/s1470-2045(07)70341-8

11. Andreyev HJ, Muls AC, Norton C, Ralph C, Watson L, Shaw C, Lindsay JO (2015) Guidance: The practical management of the gastrointestinal symptoms of pelvic radiation disease. Frontline gastroenterology 6 (1):53-72. doi:10.1136/flgastro-2014-100468

For peer review only

12. Schover LR, van der Kaaij M, van Dorst E, Creutzberg C, Huyghe E, Kiserud CE (2014) Sexual dysfunction and infertility as late effects of cancer treatment. European Journal of Cancer Supplements 12 (1):41-53. doi:http://dx.doi.org/10.1016/j.ejcsup.2014.03.004

13. Schover LR, Fife M, Gershenson DM (1989) Sexual dysfunction and treatment for early stage cervical cancer. Cancer 63 (1):204-212

14. Bergmark K, Avall-Lundqvist E, Dickman PW, Henningsohn L, Steineck G (2002) Patient-rating of distressful symptoms after treatment for early cervical cancer. Acta Obstet Gynecol Scand 81 (5):443-450

15. Dunberger G, Lind H, Steineck G, Waldenstrom AC, Onelov E, Avall-Lundqvist E (2011) Loose stools lead to fecal incontinence among gynecological cancer survivors. Acta oncologica (Stockholm, Sweden) 50 (2):233-242. doi:10.3109/0284186x.2010.535013

16. Andreyev HJ, Wotherspoon A, Denham JW, Hauer-Jensen M (2011) "Pelvic radiation disease": new understanding and new solutions for a new disease in the era of cancer survivorship. Scand J Gastroenterol 46 (4):389-397. doi:10.3109/00365521.2010.545832

17. Steineck G, Skokic V, Sjoberg F, Bull C, Alevronta E, Dunberger G, Bergmark K, Wilderang U, Oh JH, Deasy JO, Jornsten R (2017) Identifying radiation-induced survivorship syndromes affecting bowel health in a cohort of gynecological cancer survivors. PloS one 12 (2):e0171461. doi:10.1371/journal.pone.0171461

18. Lindroth T, Islind AS, Steineck G, Lundin J (2018) From narratives to numbers: Data work and patient-generated health data in consultations. Studies in Health Technology and Informatics, vol 247. doi:10.3233/978-1-61499-852-5-491

19. Cerna K, Ivarsson J, Weilenmann A, Steineck G (2019) Supporting self-management of radiation-induced bowel and bladder dysfunction in pelvic-cancer rehabilitation: An ethnographic study. Journal of clinical nursing. doi:10.1111/jocn.14849

20. Andreyev HJN, Davidson SE, Gillespie C, Allum WH, Swarbrick E (2012) Practice guidance on the management of acute and chronic gastrointestinal problems arising as a result of treatment for cancer. Gut 61 (2):179. doi:10.1136/gutjnl-2011-300563

For peer review only - http://bmjopen.bmj.com/site/about/guidelines.xhtml

For peer review only

409 21. Annon JS (1976) The PLISSIT model: A proposed conceptual scheme for the behavioral
410 treatment of sexual problems. Journal of Sex Education and Therapy 2 (1):1-15.
411 doi:10.1080/01614576.1976.11074483
412 22. Basson R, Wierman ME, van Lankveld J, Brotto L (2010) Summary of the recommendations on
413 sexual dysfunctions in women. The journal of sexual medicine 7 (1 Pt 2):314-326.
414 doi:10.1111/j.1743-6109.2009.01617.x
415 23. Carter J, Goldfrank D, Schover LR (2011) Simple Strategies for Vaginal Health Promotion in
416 Cancer Survivors. The journal of sexual medicine 8 (2):549-559. doi:10.1111/j.1743-
417 6109.2010.01988.x
418 24. DuHamel K, Schuler T, Nelson C, Philip E, Temple L, Schover L, Baser RE, Starr TD, Cannon K,
419 Jennings S, Jandorf L, Carter J (2016) The sexual health of female rectal and anal cancer survivors:
420 results of a pilot randomized psycho-educational intervention trial. Journal of cancer survivorship :
421 research and practice 10 (3):553-563. doi:10.1007/s11764-015-0501-8
422 25. Faithfull S, White I (2008) Delivering sensitive health care information: challenging the taboo of
423 women's sexual health after pelvic radiotherapy. Patient Educ Couns 71 (2):228-233.
424 doi:10.1016/j.pec.2007.12.011
425 26. Bergmark K, Åvall-Lundqvist E, Dickman PW, Henningsohn L, Steineck G (2005) Synergy
426 Between Sexual Abuse and Cervical Cancer in Causing Sexual Dysfunction. Journal of sex & marital
427 therapy 31 (5):361-383. doi:10.1080/00926230591006476
428 27. Aoki Y, Brown HW, Brubaker L, Cornu JN, Daly JO, Cartwright R (2017) Urinary incontinence
429 in women. Nature reviews Disease primers 3:17042-17042. doi:10.1038/nrdp.2017.42
430 28. Leddy LSJCUR (2018) Management of Lower Urinary Tract Symptoms After Pelvic Radiation in
431 Females. 19 (12):106. doi:10.1007/s11934-018-0848-2
432 29. Lawenda BD, Mondry TE, Johnstone PAS (2009) Lymphedema: A primer on the identification
433 and management of a chronic condition in oncologic treatment. 59 (1):8-24. doi:10.3322/caac.20001
434 30. Dunberger G, Lindquist H, Waldenstrom AC, Nyberg T, Steineck G, Avall-Lundqvist E (2013)
435 Lower limb lymphedema in gynecological cancer survivors--effect on daily life functioning.

For peer review only - http://bmjopen.bmj.com/site/about/guidelines.xhtml

Supportive care in cancer : official journal of the Multinational Association of Supportive Care in Cancer 21 (11):3063-3070. doi:10.1007/s00520-013-1879-3

31. Bakar Y, Tuğral A (2017) Lower Extremity Lymphedema Management after Gynecologic Cancer Surgery: A Review of Current Management Strategies. Annals of Vascular Surgery 44:442-450. doi:https://doi.org/10.1016/j.avsg.2017.03.197

32. Schofield P, Juraskova I, Bergin R, Gough K, Mileshkin L, Krishnasamy M, White K, Bernshaw D, Penberthy S, Aranda S (2013) A nurse- and peer-led support program to assist women in gynaecological oncology receiving curative radiotherapy, the PeNTAGOn study (peer and nurse support trial to assist women in gynaecological oncology): study protocol for a randomised controlled trial. Trials 14:39. doi:10.1186/1745-6215-14-39

33. Jefford M, Aranda S, Gough K, Lotfi-Jam K, Butow P, Krishnasamy M, Young J, Phipps-Nelson J, Russell L, King D, Schofield P (2013) Evaluating a nurse-led survivorship care package (SurvivorCare) for bowel cancer survivors: study protocol for a randomized controlled trial. Trials 14:260-260. doi:10.1186/1745-6215-14-260

34. Hofsjo A, Bohm-Starke N, Blomgren B, Jahren H, Steineck G, Bergmark K (2017) Radiotherapy-induced vaginal fibrosis in cervical cancer survivors. Acta oncologica (Stockholm, Sweden):1-9. doi:10.1080/0284186x.2016.1275778

35. Westman B, Ullgren H, Olofsson A, Sharp L (2019) Patient-reported perceptions of care after the introduction of a new advanced cancer nursing role in Sweden. European Journal of Oncology Nursing 41:41-48. doi:10.1016/j.ejon.2019.05.009

36. Andersen B, Karlsson J, Anderson B, Tewfik H (1984) Anxiety and cancer treatment: Response to stressful radiotherapy. Health Psychology 3:535-551. doi:10.1037/0278-6133.3.6.535

37. Oberoi D, White VM, Seymour JF, Miles Prince H, Harrison S, Jefford M, Winship I, Hill D, Bolton D, Kay A, Millar J, Doo NW, Giles G (2017) The influence of unmet supportive care needs on anxiety and depression during cancer treatment and beyond: a longitudinal study of survivors of haematological cancers. Supportive care in cancer : official journal of the Multinational Association of Supportive Care in Cancer 25 (11):3447-3456. doi:10.1007/s00520-017-3766-9

For peer review only - http://bmjopen.bmj.com/site/about/guidelines.xhtml

BMJ Cohort profile

38. Stubblefield MD (2017) The Underutilization of Rehabilitation to Treat Physical Impairments in Breast Cancer Survivors. 9 (9S2):S317-S323. doi:10.1016/j.pmrj.2017.05.010

39. Penalba V, Deshields TL, Klinkenberg DJSCiC (2019) Gaps in communication between cancer patients and healthcare providers: symptom distress and patients' intentions to disclose. 27 (6):2039-2047. doi:10.1007/s00520-018-4442-4

40. Rasmusson EM, Plantin L, Elmerstig E (2013) 'Did they think I would understand all that on my own?' A questionnaire study about sexuality with Swedish cancer patients. Eur J Cancer Care (Engl) 22 (3):361-369. doi:10.1111/ecc.12039

41. Bakker RM, Mens JW, de Groot HE, Tuijnman-Raasveld CC, Braat C, Hompus WC, Poelman JG, Laman MS, Velema LA, de Kroon CD, van Doorn HC, Creutzberg CL, Ter Kuile MM (2017) A nurse-led sexual rehabilitation intervention after radiotherapy for gynecological cancer. Supportive care in cancer : official journal of the Multinational Association of Supportive Care in Cancer 25 (3):729-737. doi:10.1007/s00520-016-3453-2

42. WHO (2006) Sexual health and Sexuality, definition. http://www.who.int/reproductivehealth/topics/sexual_health/sh_definitions/en/.

43. Chapman CH, Heath G, Fairchild P, Berger MB, Wittmann D, Uppal S, Tolpadi A, Maturen K, Jolly SJJoCR, Oncology C (2019) Gynecologic radiation oncology patients report unmet needs regarding sexual health communication with providers. 145 (2):495-502. doi:10.1007/s00432-018-2813-3

44. Hordern AJ, Street AF (2007) Communicating about patient sexuality and intimacy after cancer: mismatched expectations and unmet needs. The Medical journal of Australia 186 (5):224-227

45. Concato J, Shah N, Horwitz RI (2000) Randomized, Controlled Trials, Observational Studies, and the Hierarchy of Research Designs. 342 (25):1887-1892. doi:10.1056/nejm200006223422507

46. Althubaiti A (2016) Information bias in health research: definition, pitfalls, and adjustment methods. J Multidiscip Healthc 9:211-217. doi:10.2147/JMDH.S104807

47. Steineck G, Bergmark K, Henningsohn L, al-Abany M, Dickman PW, Helgason A (2002) Symptom documentation in cancer survivors as a basis for therapy modifications. Acta oncologica (Stockholm, Sweden) 41 (3):244-252

For peer review only

48. Steineck G, Hunt H, Adolfsson J (2006) A hierarchical step-model for causation of bias-evaluating cancer treatment with epidemiological methods. Acta oncologica (Stockholm, Sweden) 45 (4):421-429. doi:10.1080/02841860600649293

For peer review only

For peer review only - http://bmjopen.bmj.com/site/about/guidelines.xhtml

BMJ Open

Cohort profile: An observational longitudinal data collection of health aspects in a cohort of female cancer survivors with a history of pelvic radiotherapy, a population-based cohort in the Western Region of Sweden

Journal:	*BMJ Open*
Manuscript ID	bmjopen-2021-049479.R1
Article Type:	Cohort profile
Date Submitted by the Author:	15-Jun-2021
Complete List of Authors:	Åkeflo, Linda; University of Gothenburg Sahlgrenska Academy, Department of Oncology<br>Dunberger, Gail; Ersta Sköndal Bräcke högskola - Campus Ersta, Department of health Care Sciences<br>Elmerstig, Eva; Malmö Universitet, Centre for Sexology and Sexuality studies<br>Skokic, Viktor; University of Gothenburg Sahlgrenska Academy, Department of Oncology<br>Steineck, Gunnar; University of Gothenburg Sahlgrenska Academy, Department of Oncology<br>Bergmark, Karin; University of Gothenburg Sahlgrenska Academy, Department of Oncology
**Primary Subject Heading**:	Oncology
Secondary Subject Heading:	Oncology
Keywords:	Gastrointestinal tumours < ONCOLOGY, Gynaecological oncology < ONCOLOGY, Radiation oncology < RADIOTHERAPY

SCHOLARONE™
Manuscripts

I, the Submitting Author has the right to grant and does grant on behalf of all authors of the Work (as defined in the below author licence), an exclusive licence and/or a non-exclusive licence for contributions from authors who are: i) UK Crown employees; ii) where BMJ has agreed a CC-BY licence shall apply, and/or iii) in accordance with the terms applicable for US Federal Government officers or employees acting as part of their official duties; on a worldwide, perpetual, irrevocable, royalty-free basis to BMJ Publishing Group Ltd ("BMJ") its licensees and where the relevant Journal is co-owned by BMJ to the co-owners of the Journal, to publish the Work in this journal and any other BMJ products and to exploit all rights, as set out in our licence.

The Submitting Author accepts and understands that any supply made under these terms is made by BMJ to the Submitting Author unless you are acting as an employee on behalf of your employer or a postgraduate student of an affiliated institution which is paying any applicable article publishing charge ("APC") for Open Access articles. Where the Submitting Author wishes to make the Work available on an Open Access basis (and intends to pay the relevant APC), the terms of reuse of such Open Access shall be governed by a Creative Commons licence – details of these licences and which Creative Commons licence will apply to this Work are set out in our licence referred to above.

Other than as permitted in any relevant BMJ Author's Self Archiving Policies, I confirm this Work has not been accepted for publication elsewhere, is not being considered for publication elsewhere and does not duplicate material already published. I confirm all authors consent to publication of this Work and authorise the granting of this licence.

For peer review only

BMJ Cohort profile

Cohort profile: An observational longitudinal data collection of health aspects in a cohort of female cancer survivors with a history of pelvic radiotherapy, a population-based cohort in the Western Region of Sweden

Linda Åkeflo[a]*, Gail Dunberger[b] , Eva Elmerstig[c], Viktor Skokic[a], Gunnar Steineck[a] , Karin Bergmark[a]

[a]*Department of Clinical Cancer Epidemiology, Department of Oncology, Institute of Clinical Science, The Sahlgrenska Academy, University of Gothenburg, Gothenburg, Sweden;* [b]*Department of Health Care Sciences, Ersta Sköndal Bräcke University College, Stockholm, Sweden;* [c]*Centre for Sexology and Sexuality studies, Malmö University, Malmö, Sweden,*

*Correspondence: L. Åkeflo, Department of clinical Cancer Epidemiology, Department of Oncology, Sahlgrenska Academy at University of Gothenburg, 413 45 Gothenburg, Sweden. Tel: +46 732 017972. Fax: +46 31 823931. E-mail: linda.akeflo@vgregion.se

For peer review only - http://bmjopen.bmj.com/site/about/guidelines.xhtml

BMJ Cohort profile

ABSTRACT

Purpose: The study "Health among women after pelvic radiotherapy" was conducted in response to the need for more advanced and longitudinal data concerning long-term radiotherapy-induced late effects and chronic states among female cancer survivors. The objective of this paper is to detail the cohort profile and the study procedure in order to provide a sound basis for future analyses of the study cohort.

Participants: Since 2011, and still currently ongoing, participants have been recruited from a population-based study cohort including all female patients with cancer, over 18 years of age, treated with pelvic radiotherapy with curative intent at Sahlgrenska University Hospital in Gothenburg, in the Western Region of Sweden, which covers 1.7 million of the Swedish population. The dataset presented here consists of baseline data provided by 605 female cancer survivors and three-month follow-up data from 260 individuals with gynaecological-, rectal- or anal cancer, collected over a six-year period.

Findings to date: Data has been collected from 2011 onwards. To date, three studies have been published using the dataset reporting long-term radiation-induced intestinal syndromes and late adverse effects affecting sexuality, the urinary tract, the lymphatic system, and physical activity. These projects include the evaluation of interventions developed by and provided in a nurse-led clinic.

Future plans: This large prospective cohort offers the possibility to study health outcomes in female pelvic cancer survivors undergoing a rehabilitation intervention in a nurse-led clinic, and to study associations between demographics, clinical aspects and long-term late effects. Analysis focusing on the effect of the interventions on sexual health aspects, pre- and post-interventions, is currently ongoing. The cohort will be expanded to comprise the entire data collection from 2011-2020, including baseline data and data from three-month and one-year follow-ups after interventions. The data will be used to study conditions and treatment-induced late effects pre- and post-intervention.

Keywords: *Female cancer survivors, Treatment-induced late effects, Pelvic radiotherapy, Cancer rehabilitation, Nurse-led clinic*

Strengths and limitations

- The major strength of the study is the large representative population-based cohort (n=605)
- Uses a large longitudinal dataset consisting of female cancer survivors' entries from six months to several years post-pelvic radiotherapy treatment
- Highlighting treatment-induced cancer survivorship diseases and chronic states improves the possibilities for developing further effective treatments

For peer review only - http://bmjopen.bmj.com/site/about/guidelines.xhtml

- One limitation of the study is the reliance on self-reported data, which has the potential for response bias
- The interventions that were provided varied over the course of the study, which is considered a potential limitation

Introduction

An increasing number of individuals now live longer after a cancer diagnosis than was previously common, and more and more patients are living with treatment-induced late effects, treatment-induced cancer survivorship diseases, and survivorship chronic states [1-3]. Pelvic radiotherapy affects the intestinal and urinary tracts, the lymphatic system, and sexual health [4-10]. In a video at https://www.jostrust.org.uk/video/letstalk-pelvic-radiation-disease-after-cervical-cancer, some of more than 20 million cancer survivors in Europe describe how radiation-induced impaired intestinal health affects them. In Sweden, approximately 3,500 women are diagnosed with pelvic cancer (gynaecological, anal, or rectal cancer) every year [11]. In 2011, we started a nurse-led clinic focusing on treatment-induced late effects among pelvic cancer survivors with the intent of developing self-care strategies and treatments. We have previously reported on the benefits of the clinic regarding the resulting improved quality of life and psychosocial wellbeing among female pelvic cancer survivors [12].

We aim to describe a population-based cohort consisting of female cancer survivors treated with pelvic radiotherapy using a dataset from a longitudinal study that has been ongoing since 2011. A further aim is to describe the data collection procedure, the interventions provided in a nurse-led clinic, and the characteristics of the study cohort. In addition, a few basal empirical results will be presented to illustrate the study cohort. The objective of this paper is to detail the cohort profile and the study procedure in order to provide a sound basis for future analyses in the study cohort.

Cohort description

Setting

In 2009, the Swedish government proposed a new regulation: "A National Cancer Strategy" (SOU2009:1) [13]. In collaboration with the Swedish county councils and regions, six Regional Cancer Centres (RCC) were

For peer review only - http://bmjopen.bmj.com/site/about/guidelines.xhtml

For peer review only

established. Based on the national strategy proposals, the RCC in western Sweden financed a nurse-led clinic at Sahlgrenska University Hospital. The nurse-led clinic was founded in 2011 by a senior consultant physician who has a PhD and is specialised in gynaecological oncology, and an oncology nurse with a PhD in oncology. The team currently consists of three clinical oncology nurses who are specially trained and clinically specialised in understanding and addressing issues concerning pelvic cancer survivorship late effects. The cancer survivors receive education about radiotherapy-induced late effects, basic anatomy and physiology and, together with the nurse, decide on supportive care actions regarding medication, nutrition, and coping with sexual, psychological, and social challenges. In cases with more specific needs, intercurrent diseases, or a suspected recurrence of cancer, the patient meets the senior medical consultant who has the primary medical responsibility for the clinic. The senior medical consultant meets regularly with the team and discusses plans for patients' medication with the staff member who will issue the prescriptions. The healthcare program developed in the clinic is based on programs developed by others and on our previous studies in this area [4,14-20].

Patient and Public Involvement

Patients and/or the public are not involved in the design, conducting, reporting, or dissemination plans in this research. Results from future studies from the cohort will be submitted for publication in peer-reviewed journals and presented at relevant conferences.

Participants

Study participants are recruited from two different cohorts: 1) a population-based study cohort group including all female patients with cancer treated with curative intent from 2007 onwards who are continuously identified from medical records at Sahlgrenska University Hospital in Sweden and 2) a referred patient group including all female patients with cancer referred to the rehabilitation clinic. Inclusion criteria are female cancer survivors who completed pelvic radiotherapy at least six months prior to inclusion. Exclusion criteria are cancer recurrence, inability to comprehend or answer the questionnaire, and poor proficiency in the Swedish language. Data are collected from female cancer survivors with a history of pelvic radiotherapy in western Sweden, an area that includes 20 percent of the Swedish population. The tradition of collecting registry data in Sweden offers good opportunities for studying cancer survivors without selection-induced problems. The dataset

For peer review only - http://bmjopen.bmj.com/site/about/guidelines.xhtml

contains information about the pre-intervention and post-intervention needs of women who received pelvic radiotherapy at Sahlgrenska University Hospital between 2007 and 2016. Female cancer survivors referred to the nurse-led clinic are also invited to participate in the study.

Invited women

An introductory letter is sent to eligible study participants. Shortly after, a research secretary phones them and gives oral information about the study, asks if they are willing to participate and, if so, asks them to give permission to be sent a written informed consent and the questionnaire. When the baseline questionnaire is returned, the participant receives an invitation to attend the nurse-led clinic where individual healthcare interventions are conducted. The code number from the questionnaire (followed throughout the data collection) is entered into the patient-database FileMaker Pro17 Advanced® that was specifically designed to suit the study. The study participant receives a follow-up questionnaire three and twelve months after the completed intervention. The research secretary keeps track of each patient and, if case questionnaires are delayed or missing, sends two reminders at time points determined in advance, see Figure 1.

Referred patients

Patients referred from oncology healthcare providers, general practitioners, and through private referrals, who meet the inclusion criteria are also invited to participate in the study. The data collection procedure follows the same principles as for the invited women in the population-based cohort.

Study-specific questionnaires

As shown in Table 1, the baseline questionnaire consists of 175 questions, divided into eight main sections and one concluding chapter. Information is obtained about the frequency, intensity, duration, and quality of each symptom, and the degree of distress it causes the patient. An example of one question is: "How many times per week (approximately) have you had bowel movements during the past six months?" with possible answers: "About every other day", "Less than once a day", "Once a day", "Up to twice a day", "2-3 times per day", "3-4

For peer review only - http://bmjopen.bmj.com/site/about/guidelines.xhtml

times per day", or "5 times or more per day". To facilitate statistical analyses, the answers are coded into values, ranked, and placed into groups. Variables such as age and number of children are categorised into groups.

Table 1. The study-specific baseline questionnaire, divided into eight sections and one concluding chapter

Section	Question areas
Sociodemographic	Gender<br>Age<br>Marital status<br>Number of children<br>Level of education<br>Employment status
Quality of life and wellbeing	Quality of life<br>Depression<br>Worry<br>Anxiety
Body perception and self-image	Femininity<br>Self-esteem<br>Fertility<br>Childbirth<br>Vaginal and perianal injury related to childbirth or physical trauma
Intestinal and defecation habits	Loose stools<br>Faecal incontinence<br>Urgency to defecate<br>Excessive gas<br>Abdominal pain<br>Having a stoma<br>Medical treatment related to intestinal symptoms
Micturition habits and urinary tract symptoms	Urinary frequency<br>Urgency to urinate<br>Nocturia<br>Having a urinary catheter<br>Medical treatment due to symptoms
Sexual health	Menopause<br>Use of systemic hormone replacement therapy<br>Use of topical oestrogen<br>Impaired lubrication<br>Vaginal shortness<br>Vaginal inelasticity<br>Dyspareunia
Sexual abuse	Experience of sexual abuse<br>Experience of sexual harassment<br>Age when experienced sexual abuse or harassment<br>Extent to which the experience affects sexual life
Lymphoedema	Heaviness in legs, genitals and abdomen<br>If diagnosed with lymphoedema, current lymphoedema treatment
Concluding chapter	Self-reported needs<br>Invite to visit the nurse-led clinic for assessment and counselling

Several sections allow free comments

The questionnaire also addresses the extent to which the symptoms affect social functioning. In the concluding chapter, the participants are invited to visit the nurse-led clinic. Some questions require the patients to rank their

For peer review only

For peer review only - http://bmjopen.bmj.com/site/about/guidelines.xhtml

most distressing symptoms. In the follow-up questionnaires, the study participants' health status and symptoms are measured, and the interventions conducted at the nurse-led clinic are evaluated.

Diagnostic and alleviative means

The questionnaire serves as a basis for the interventions conducted at the first visit to the clinic. The patient's current health status reveals the areas that need to be focused on. Current symptoms and self-care strategies are discussed. New strategies are then suggested and decided on together with the patient. The duration of the contact and the number of follow-ups varies from one month to one year depending on the symptoms and the effect of treatments and interventions.

Intestinal health

The assessment is based on five syndromes: urgency to defecate, faecal leakage, excessive gas production, excessive mucus discharge, and blood discharge [21]. Symptoms, such as leakage of mucus and blood, abdominal bloating, signs of bacterial overgrowth, and bile salt malabsorption, are also assessed. Diagnostic means, including blood tests such as electrolytes, blood counts, vitamin B12 and serum magnesium, are taken when indicated. An algorithm developed by the research group serves as a guide in the clinical setting [12]. A mobile application has also been developed that was specially designed for radiation-induced intestinal syndromes and which can show graphs of the variation in intestinal health over time. The application serves as a complement to existing methods of patients' self-assessment and self-management, and helps to support the nurse's and patient's conversation and decision-making [22,23]. The objective of the interventions is to restore or improve intestinal health and includes medical treatment, pelvic floor muscle training, and techniques using cognitive training to control intestinal function. Details of these interventions can be accessed elsewhere [12,24].

Sexual health

The PLISSIT-model (Permission, Limited Information, Specific Suggestions, and Intensive Therapy) developed by Annon [25] is used to address sexual health concerns. The first three levels in the model are helpful for most patients. When specific suggestions are not sufficient, the patient is recommended to take part in Intensive

For peer review only - http://bmjopen.bmj.com/site/about/guidelines.xhtml

Therapy, which is sometimes given in the clinical setting and other times with a sexologist or psychotherapist after a referral is sent. Female sexual dysfunction is a complex condition, including diverse aspects, definitions, and classifications [26]. To assess and treat sexual dysfunction, previous sexual practices and experiences are discussed, with particular attention given to patients' integrity, special needs, and preferences. Structured information about common physical late effects involving vaginal changes and sexual problems is provided as well as information concerning psychological issues influencing sexual health; all information given is in accordance with evidence-based knowledge and practice in the field [4,17,27-29,1]. Patients receive information about and instructions on the use of vaginal dilator therapy and topical oestrogen, and they are also given guidance and suggestions related to lack of sexual desire. When requested, specific sexual devices, films, and literature are suggested to encourage and help the patient to regain sexual function, which for some patients is helpful in managing issues of body image perception, self-esteem, and intimacy.

Sexual abuse

Patients with a history of sexual abuse have an increased risk of developing sexual problems after cancer treatment, and a large proportion of women with cervical cancer and those experiencing dyspareunia post-cancer treatment have been sexually abused [30]. In cases where this is reported, experiences of sexual abuse are carefully and sensitively discussed with the patient and, in some cases, this may lead to referral to a psychologist or therapist.

Urinary tract health

Symptoms of urgency, nocturia, urinary retention, urinary tract infection, and pelvic pain are addressed. The diagnostic methods used include evaluations of urinary frequency and of symptoms, which are divided into irritative symptoms, obstructive symptoms, and bleeding. Patients can keep a 72-hour voiding diary, including urine volume and fluid intake, which is a helpful resource for this discussion. Guidance and recommendations are given about topical oestrogen, pelvic floor muscle training, medical treatments, self-care modifications, and behavioural interventions, all in accordance with previous evidence-based knowledge [31,32]. In cases of severe urinary tract symptoms, referrals are sent to a urotherapist, physiotherapist, or urologist.

For peer review only - http://bmjopen.bmj.com/site/about/guidelines.xhtml

Lymphoedema

Lower limb lymphoedema is a common non-curable chronic complication with multifactorial pathophysiology [33]. Early detection and treatment of lymphoedema is essential in order to prevent complications. Self-reported or objectively assessed swelling or heaviness in the lower limbs leads to referral for lymphatic therapy. The primary therapy is the use of individually tailored compression garments and second-line therapy consists of manual lymphatic drainage with intermittent use of pumps and other self-care strategies [34,35].

Statistical analyses

Initially, data from the questionnaires were entered into EpiDataSoftware® version 3.1 (EpiData Association). Since only one answer to each question is allowed, in the event of two answers the procedure was to alternate entering the first provided answer and the second provided answer. In order to facilitate the analysis of responses to the open-ended questions, the answers to these questions were transcribed in Microsoft Word (2016). R version 3.5.2. was used for statistical analysis of the data. The results will be reported in means, medians, and percentages.

Ethics

All procedures in the study, which involved human participants, were in accordance with the ethical standards of the Regional Ethics Review Board in Gothenburg (D 686-10) and with the 1964 Declaration of Helsinki and its later amendments. The research was approved by the Regional Ethics Review Board in Gothenburg (D 686-10), Gothenburg University. Written informed consent was obtained from all individual participants included in the study.

Findings to date

Three studies based on the cohort have been published [12,36,37]. Two papers focused on gastrointestinal side effects and one on sexual health aspects. It was found that the majority of the women reported a change in bowel habits and, in almost half of them, the effect was considerable [12]. Women with faecal leakage were less likely to practise physical activity than survivors without leakage, and survivors who practised weekly physical

For peer review only - http://bmjopen.bmj.com/site/about/guidelines.xhtml

activity experienced better quality of life and were less frequently in a depressed mood than women not physical active [36]. Furthermore, a statistically significant increase in psychological distress and sexual health impairment was found among women with a history of sexual abuse compared to women without such experience [37].

Characteristics of study participants

From January 2011 to June 2017, we identified a total of 791 patients in the population-based cohort who met the inclusion criteria. Of the total sample, 684 (86%) individuals gave oral consent to participate in the study and of these, 464 (68%) completed the baseline questionnaire.

During the same period, 184 referred patients met the inclusion criteria and were invited to participate in the study. Of those invited to participate, 141 (76.6%) completed the baseline questionnaire and of these, 131 (92%) took part in interventions.

The responses and completeness of the questionnaires from both study groups are shown in the schematic diagram, see Figure 2. In Table 2, we present the baseline characteristics and demographics in the dataset, which consists of 605 participants: 464 (76.7%) from the invited cohort and 161 (23.3%) from the referred cohort.

Table 2. Characteristics of the study participants

Variable	Total	Invited	Referred
**Participants, n (%)**	605	464 (76.7)	141 (23.3)
**Cancer type, n (%)**			
Endometrial cancer	216 (35.7)	181 (39.0)	35 (24.8)
Cervical cancer	132 (21.8)	80 (17.2)	52 (36.9)
Ovarian cancer	2 (0.3)	1 (0.2)	1 (0.7)
Vaginal cancer	5 (0.8)	3 (0.6)	2 (1.4)
Vulvar cancer	21 (3.5)	19 (4.1)	2 (1.4)
Anal cancer	80 (13.2)	58 (12.5)	22 (15.6)
Rectal cancer	145 (24.0)	122 (26.3)	23 (16.3)
Other	4 (0.7)		4 (2.8)
**Age in years**			
Mean	64.5	66.5	57.6
SD	12.6	11.5	13.6
Missing, (%)	11	7 (1.5)	4 (2.8)
**Years since radiotherapy, grouped**			
0	35 (5.8)	6 (1.3)	29 (20.6)
1	219 (36.1)	166 (35.8)	53 (37.6)
2	98 (16.1)	86 (18.5)	12 (8.5)
3	139 (23.0)	131 (28.2)	8 (5.7)
≥4	105 (17.3)	69 (14.9)	36 (25.5)
Missing	9 (1.5)	6 (1.3)	3 (2.1)
Mean	2.6	2.2	3.9
SD	3.4	1.2	6.7

For peer review only

For peer review only - http://bmjopen.bmj.com/site/about/guidelines.xhtml

**Cancer treatment, n (%)**			
External radiotherapy, only	145 (24.0)	101 (21.8)	44 (31.2)
External radiotherapy and brachytherapy	20 (3.3)	16 (3.4)	4 (2.8)
External radiotherapy, brachytherapy and surgery	180 (29.7)	157 (33.8)	23 (16.3)
External radiotherapy and surgery	260 (43.0)	190 (40.9)	70 (49.6)
**Marital status, n (%)**			
Married or living with a partner	402 (66.4)	309 (66.6)	93 (66.0)
Widow	70 (11.6)	59 (12.7)	11 (7.8)
Has a partner but lives alone	30 (5.0)	15 (3.2)	15 (10.6)
Single	102 (17.0)	80 (17.2)	22 (15.6)
Missing	1 (0.2)	1 (0.2)	
**Education level, n (%)**			
Elementary school	173 (29.1)	150 (32.3)	23 (16.3)
Secondary school	227 (38.2)	169 (36.4)	58 (41.1)
College/University	194 (32.0)	135 (32.3)	59 (41.8)
Missing	11 (1.8)	10 (2.2)	1 (0.7)
**Employment status, n (%)**			
Student	4 (0.7)	3 (0.6)	1 (0.7)
Unemployed job seeker	12 (2.0)	10 (2.2)	2 (1.4)
Employed	162 (27.0)	116 (25.0)	46 (32.6)
Housewife	4 (0.7)	2 (0.2)	2 (1.4)
On sick leave	54 (9.0)	17 (3.7)	37 (26.2)
Disability pension	35 (5.7)	26 (5.6)	9 (6.4)
Retired	328 (54.7)	284 (61.2)	44 (31.2)
Missing	6 (1.0)	6 (1.3)	
**Resident, n (%)**			
In a big city	182 (30.6)	131 (28.2)	51 (36.2)
In a small or medium-sized city	309 (50.2)	244 (52.6)	61 (43.3)
In the countryside	116 (18.9)	87 (18.8)	29 (20.6)
Missing	2 (0.3)	2 (0.4)	
**Smoking, n (%)**			
Does not smoke	448 (74.0)	339 (73.1)	109 (77.3)
Smokes	67 (11.0)	52 (11.2)	17 (12.1)
Missing	88 (14.5	73 (15.7)	15 (10.6)

N (number) and proportion (%) of women is presented. SD = Standard Deviation

The majority of the study participants had a history of gynaecological cancer and had been treated with radiotherapy in combination with surgery. In the population-based study cohort, the mean age was higher and twice as many were retired compared to the referred group. In the referred group, twice as many were on sick leave. In total, 379 (63%) of the 605 study participants agreed to visit the clinic, see Table 3. Sixty-seven (14.4%) of the women in the population-based study group who declined to visit the clinic reported that they had radiotherapy-induced late effects.

Table 3. Number (N) and proportion (%) of study participants who agreed to visit the clinic

	Total N (%)	Invited N (%)	Referred N (%)
No, I have no late effects and do not need to visit the clinic	108 (17.8)	107 (23.1)	1 (0.7)

For peer review only - http://bmjopen.bmj.com/site/about/guidelines.xhtml

For peer review only

No, I have late effects, but I do not want to visit the clinic	71 (11.7)	67 (14.4)	4 (2.8)
Yes, I want to visit the clinic	379 (62.6)	248 (53.4)	131 (92.9)
Missing	47 (7.8)	42 (9.1)	5 (3.5)

N (number) and proportion (%) of women is presented

Strengths and limitations

In this paper, we describe the population-based cohort consisting of female cancer survivors treated with pelvic radiotherapy in the western region of Sweden. The data collection procedure, the interventions provided, and the characteristics of the study cohort are also outlined. In addition, a few basal empirical results are reported to illustrate the study population. The major strength of the study is the large population-based cohort, since it creates the possibility of studying cancer survivors without selection-induced problems and makes it representative of the reference population consisting of an increasing number of female cancer survivors treated with pelvic radiotherapy. The longitudinal study design enables future investigations of long-term treatment-induced late effects, diseases, and chronic states. Moreover, it will be possible to evaluate the long-term outcomes of interventions and the treatments provided.

The data collection has generated a large dataset consisting of patient entries from six months to several years post-pelvic radiotherapy treatment. Over a period of six and a half years, almost 1,000 female pelvic cancer survivors have been invited to participate in the study; the dataset consists of 605 cancer survivors. In the population-based cohort group, 68% completed a baseline questionnaire. Treatment and interventions concerning physical, psychosocial, and sexual issues were offered to patients in both the population-based cohort and the referred cohort, and these were evaluated. As shown in Table 3, 53.4% of the study participants in the population-based cohort agreed to visit the clinic, which may indicate the proportion of cancer survivors with unmet needs. Improved self-care strategies, increased clinical knowledge, and developments in technology currently provide healthcare professionals with possible methods to help cancer survivors manage and treat the late effects of pelvic radiotherapy treatment. We believe that highlighting treatment-induced cancer survivorship diseases and chronic states may increase the likelihood of further effective treatments being developed.

For peer review only

For peer review only - http://bmjopen.bmj.com/site/about/guidelines.xhtml

Cancer survivorship issues have advanced from being neglected to gradually being given increasingly greater attention in healthcare practice as well as in research. This is reflected by the development of national guidelines and the organisation of national and international scientific conferences and meetings in this subject area. Efforts are being made to both understand how treatment-induced late effects manifest in pelvic cancer survivors and to find strategies to deal with these late effects. Twenty years have passed since researchers within our team [4] first observed that patients with gynaecological cancer suffered from vaginal changes affecting their sexual health. More recently, we have identified five syndromes impairing pelvic cancer survivors' intestinal health. Results published in 2017 [21] simplify the search for ways to prevent, manage, and help minimise the occurrence and intensity of survivorship diseases. One promising ongoing study being conducted by Schofield et al in Australia is evaluating a care program similar to ours [38,39]. Furthermore, in 2015 Andreyev et al [15] published a guide for the management of intestinal problems, including an algorithm that, in our opinion, is a useful tool for clinicians. However, the pathophysiological changes, which are described in numerous previous studies, are not yet fully understood [19-21]. Inflammatory and fibrotic processes in the gut wall are probably of importance in explaining the various symptoms. The processes may relate to both the intestinal tract and other organs located in the pelvis. Hofsjö et al in our research group [40] recently studied vaginal changes and found morphological explanations for changes in the vaginal wall. Biopsies from the vaginal connective tissue affected by radiotherapy showed dense collagen and entangled elastin fibres, a finding that may explain common symptoms such as reduced vaginal elasticity during intercourse, reduced lubrication, and dyspareunia.

In Sweden, promising steps are being taken towards developing a program for national coordination of cancer rehabilitation practice. Cerna et al [23], in a study concerning self-management from an educational perspective, observed that patients and nurses can together create tailor-made solutions. The nurses focus on encouraging the patients to self-reflect and on maintaining the patient's motivation to continue to engage in self-care. The ongoing establishment of oncology nurse navigators [41] is also an initiative that has the potential to increase the supportive care given to cancer survivors. In the 1980s, research in the psychological field [42] showed that patients have a high risk of anxiety related to oncology treatment. Recent studies report that patients have a lower risk of future anxiety and depression when their needs are addressed during treatment [43,44]. In our opinion, when planning future follow-up in clinical cancer care, these findings need to be taken into account,

For peer review only - http://bmjopen.bmj.com/site/about/guidelines.xhtml

For peer review only

irrespective of the patient's cancer diagnosis. We suggest that, in the future, healthcare should provide advanced specialist expertise in the management of severe treatment-induced late effects.

Sexual concerns are generally not addressed or discussed as much as patients would like [45], and patients generally wait for healthcare professionals to raise the subject [46]. In our clinical setting, sexual health conversations are integrated into the clinical work, routines for assessment and treatment have been created, and it becomes clearer when a patient should be referred to specialists and sexologists. The PLISSIT-model [25] has been used for addressing sexual health concerns in both our own clinical setting and in that of others. Clinical experience shows that sexual function might improve and even return to pre-diagnosis level through frequent clinical follow-up, which is consistent with results from previous studies [47]. Parts of WHO's definition of sexual and reproductive health state that: "sexual health requires a positive and respectful approach to sexuality and sexual relationships" [48], so healthcare professionals need to be able to talk openly to patients about sexuality. It has been suggested that healthcare professionals should actively engage in training to improve their communication skills in order to overcome common communication barriers [49,50]. Our clinical experience has shown that several patients have felt relieved when being able to speak openly about sexual issues as well as other private health concerns. The baseline questionnaire seems to serve as a therapeutic tool that can be followed up in counselling.

One could argue that the observational study design is a limitation in this study and that a randomised clinical trial might provide data that are more reliable. However, for ethical reasons, we found it necessary to offer all patients in the population the best available intervention. Hence, we considered that the observational study design was best suited to this purpose, a design previously shown to produce reasonably useful results [51].

One important limitation of the study is the reliance on self-reported data, which has the potential for response bias. However, we considered self-reported data to provide a wider range of responses than data collected using other data collection instruments [52]. To avoid information-related problems, we took advance preventive action by using questionnaires based on the clinometric method that we have introduced, developed, used, and described in previous research projects [4,6,53,5]. We employed epidemiological methods, introduced into the cancer survivorship field by a hierarchical step-model, to manage bias and confounding [54]. This method was considered appropriate to measure causal relationship, and to examine different symptoms and characteristics in

For peer review only - http://bmjopen.bmj.com/site/about/guidelines.xhtml

treatment-induced side effects. In short, the method comprises a quantitative pre-phase of semi-structured interviews with persons suitable for the study. Thereafter, a face-to-face-validation is conducted to ensure satisfactory internal consistency.

The wide range in length of time since completion of treatment may be considered a weakness in the study; however, this will probably also facilitate measurement of symptom progression in future analyses. Another potential limitation is that the interventions provided varied over the course of the study due to the increase in both ours and others' understanding of the complexity and pathophysiological mechanisms of symptoms and late effects. The minor changes in the interventions during the study need to be considered in future analysis. The results may also be affected by non-participation and loss to follow-up. We can only speculate about the reasons for these, such as patients not having time, not feeling motivated, or being unwilling to recall their previous cancer experience. Possible reasons for patients declining an offer to visit the clinic despite having troublesome symptoms could be due to the symptoms being too severe to enable travel to the clinic. Age-related problems and long-distance transportation can be other reasons. The data collected from the referred patients will allow analysis of the prevalence of symptoms and unmet needs observed by other healthcare providers. Since the data were collected in Sweden, we do not know to what extent our analysis will be applicable to other populations.

Through frequent lectures for patients and healthcare professionals, and the implementation of treatment strategies directly to patients, we apply the knowledge we have acquired and use it in the clinic and with the cancer survivors themselves. It is also worth mentioning that our rehabilitation clinic serves as a model for similar clinics in other regions of Sweden that have been established as part of the national strategy financed by the Swedish government. The current nurse-led clinic may serve as the beginning of a future tertiary centre to develop interventions and treatments for cancer survivors.

The extent to which the interventions provided in the individualised nurse-led rehabilitation might improve health in female cancer survivors treated with pelvic radiotherapy is currently unclear. The dataset from the 3-month follow-up questionnaire was prepared and used in recently published studies, while preparation of the

For peer review only - http://bmjopen.bmj.com/site/about/guidelines.xhtml

dataset from the one-year follow-up questionnaire is ongoing. To the best of our knowledge, our study cohort is one of only a few published population-based cohorts of female pelvic cancer survivors with treatment-induced late effects receiving individualised interventions with a focus on physical and sexual health after radiotherapy. The interventions developed, outlined, and provided will hopefully contribute to further development of evidence-based management strategies in pelvic cancer rehabilitation and will be reported in future papers.

Collaboration

Requests concerning possible specific research projects and collaborative work are encouraged and can be addressed to the corresponding author.

Acknowledgements

We thank all the women participating in this study, and also oncology nurses Lisen Heden and Karin Gustafsson, and assistant Elisabeth Bystedt at BäckencancerRehabiliteringen, Sahlgrenska University Hospital for their skilful work with study inclusion and study interventions. We also thank medical student Amanda Arnell for assistance with preparing the dataset and the Regional Cancer Centre of Västra Götaland and Västra Götaland County Council for financially supporting BäckencancerRehabiliteringen at Sahlgrenska University Hospital, Gothenburg, Sweden.

Footnotes

Author's contributions:

1. Guarantor of integrity of the entire study: Gunnar Steineck
2. Study concepts and design: Karin Bergmark, Gunnar Steineck, Gail Dunberger
3. Data analysis: Linda Akeflo, Gail Dunberger, Karin Bergmark, Eva Elmerstig, Gunnar Steineck
4. Statistical analysis: Viktor Skokic
5. Manuscript preparation: Linda Akeflo, Karin Bergmark, Gail Dunberger, Eva Elmerstig
6. Manuscript editing: Linda Akeflo

All authors have read and approved the final manuscript.

For peer review only

For peer review only - http://bmjopen.bmj.com/site/about/guidelines.xhtml

Funding

This research received no specific grant from any funding agency in the public, commercial or not-for-profit sectors.

Competing interests

The authors declare that they have no conflicts of interest. The authors have full control of all primary data and agree to allow the journal to review the data if requested.

Patient consent for publication:

Not applicable.

Provenance and peer review:

Not commissioned; externally peer reviewed.

Data availability statement:

Data are available on reasonable request.

For peer review only

Abbreviations

N	Number
PLISSIT	Permission, Limited Information, Specific Suggestions and Intensive Therapy
RCC	Regional Cancer Centre
SD	Standard Deviation
SOU	Statens Offentliga Utredningar (Swedish: National Public Inquiries)
WHO	World Health Organization

References

1. Allemani C, Matsuda T, Di Carlo V, Harewood R, Matz M, Niksic M, Bonaventure A, Valkov M, Johnson CJ, Esteve J, Ogunbiyi OJ, Azevedo ESG, Chen WQ, Eser S, Engholm G, Stiller CA, Monnereau

A, Woods RR, Visser O, Lim GH, Aitken J, Weir HK, Coleman MP (2018) Global surveillance of trends in cancer survival 2000-14 (CONCORD-3): analysis of individual records for 37 513 025 patients diagnosed with one of 18 cancers from 322 population-based registries in 71 countries. Lancet (London, England) 391 (10125):1023-1075. doi:10.1016/s0140-6736(17)33326-3

2. Miller KD, Siegel RL, Lin CC, Mariotto AB, Kramer JL, Rowland JH, Stein KD, Alteri R, Jemal A (2016) Cancer treatment and survivorship statistics, 2016. 66 (4):271-289. doi:10.3322/caac.21349

3. Steineck G, Schmidt H, Alevronta E, Sjoberg F, Bull CM, Vordermark D (2016) Toward Restored Bowel Health in Rectal Cancer Survivors. Semin Radiat Oncol 26 (3):236-250. doi:10.1016/j.semradonc.2016.03.002

4. Bergmark K, Åvall-Lundqvist E, Dickman PW, Henningsohn L, Steineck G (1999) Vaginal Changes and Sexuality in Women with a History of Cervical Cancer. New England Journal of Medicine 340 (18):1383-1389. doi:doi:10.1056/NEJM199905063401802

5. Dunberger G, Lind H, Steineck G, Waldenstrom AC, Nyberg T, Al-Abany M, Nyberg U, Vall-Lundqvist E (2010) Self-reported symptoms of faecal incontinence among long-term gynaecological cancer survivors and population-based controls. European journal of cancer (Oxford, England : 1990) 46 (3):606-615. doi:10.1016/j.ejca.2009.10.023

6. Lind H, Waldenstrom AC, Dunberger G, al-Abany M, Alevronta E, Johansson KA, Olsson C, Nyberg T, Wilderang U, Steineck G, Avall-Lundqvist E (2011) Late symptoms in long-term gynaecological cancer survivors after radiation therapy: a population-based cohort study. British journal of cancer 105 (6):737-745. doi:10.1038/bjc.2011.315

7. Bober SL, Kingsberg SA, Faubion SS (2019) Sexual function after cancer: paying the price of survivorship. Climacteric : the journal of the International Menopause Society 22 (6):558-564. doi:10.1080/13697137.2019.1606796

8. Sekse RJT, Dunberger G, Olesen ML, Østerbye M, Seibaek L (2019) Lived experiences and quality of life after gynaecological cancer-An integrative review. Journal of clinical nursing 28 (9-10):1393-1421. doi:10.1111/jocn.14721

9. Morris KA, Haboubi NY (2015) Pelvic radiation therapy: Between delight and disaster. World journal of gastrointestinal surgery 7 (11):279-288. doi:10.4240/wjgs.v7.i11.279

10. Yoshihara M, Shimono R, Tsuru S, Kitamura K, Sakuda H, Oguchi H, Hirota A (2020) Risk factors for late-onset lower limb lymphedema after gynecological cancer treatment: A multi-institutional retrospective study. European journal of surgical oncology : the journal of the European Society of Surgical Oncology and the British Association of Surgical Oncology 46 (7):1334-1338. doi:10.1016/j.ejso.2020.01.033

11. Socialstyrelsen (2017) Statistical Database. https://www.socialstyrelsen.se/en/statistics-and-data/statistics/.

12. Dunberger G, Bergmark K (2012) Nurse-led care for the management of side effects of pelvic radiotherapy: what does it achieve? Current opinion in supportive and palliative care 6 (1):60-68. doi:10.1097/SPC.0b013e32834f6a95

13. 2009:11 S (2009) A National Strategy for the Future - Summary. doi:https://www.regeringen.se/49b6a7/contentassets/e343b40615eb46b395e5c65ca38d1337/summary-sou-200911

14. Andreyev J (2007) Gastrointestinal symptoms after pelvic radiotherapy: a new understanding to improve management of symptomatic patients. The Lancet Oncology 8 (11):1007-1017. doi:10.1016/s1470-2045(07)70341-8

15. Andreyev HJ, Muls AC, Norton C, Ralph C, Watson L, Shaw C, Lindsay JO (2015) Guidance: The practical management of the gastrointestinal symptoms of pelvic radiation disease. Frontline gastroenterology 6 (1):53-72. doi:10.1136/flgastro-2014-100468

16. Schover LR, van der Kaaij M, van Dorst E, Creutzberg C, Huyghe E, Kiserud CE (2014) Sexual dysfunction and infertility as late effects of cancer treatment. European Journal of Cancer Supplements 12 (1):41-53. doi:http://dx.doi.org/10.1016/j.ejcsup.2014.03.004

For peer review only - http://bmjopen.bmj.com/site/about/guidelines.xhtml

17. Schover LR, Fife M, Gershenson DM (1989) Sexual dysfunction and treatment for early stage cervical cancer. Cancer 63 (1):204-212
18. Bergmark K, Avall-Lundqvist E, Dickman PW, Henningsohn L, Steineck G (2002) Patient-rating of distressful symptoms after treatment for early cervical cancer. Acta obstetricia et gynecologica Scandinavica 81 (5):443-450
19. Dunberger G, Lind H, Steineck G, Waldenstrom AC, Onelov E, Avall-Lundqvist E (2011) Loose stools lead to fecal incontinence among gynecological cancer survivors. Acta oncologica (Stockholm, Sweden) 50 (2):233-242. doi:10.3109/0284186x.2010.535013
20. Andreyev HJ, Wotherspoon A, Denham JW, Hauer-Jensen M (2011) "Pelvic radiation disease": new understanding and new solutions for a new disease in the era of cancer survivorship. Scandinavian journal of gastroenterology 46 (4):389-397. doi:10.3109/00365521.2010.545832
21. Steineck G, Skokic V, Sjoberg F, Bull C, Alevronta E, Dunberger G, Bergmark K, Wilderang U, Oh JH, Deasy JO, Jornsten R (2017) Identifying radiation-induced survivorship syndromes affecting bowel health in a cohort of gynecological cancer survivors. PloS one 12 (2):e0171461. doi:10.1371/journal.pone.0171461
22. Lindroth T, Islind AS, Steineck G, Lundin J (2018) From narratives to numbers: Data work and patient-generated health data in consultations. Studies in Health Technology and Informatics, vol 247. doi:10.3233/978-1-61499-852-5-491
23. Cerna K, Ivarsson J, Weilenmann A, Steineck G (2019) Supporting self-management of radiation-induced bowel and bladder dysfunction in pelvic-cancer rehabilitation: An ethnographic study. Journal of clinical nursing. doi:10.1111/jocn.14849
24. Andreyev HJN, Davidson SE, Gillespie C, Allum WH, Swarbrick E (2012) Practice guidance on the management of acute and chronic gastrointestinal problems arising as a result of treatment for cancer. Gut 61 (2):179. doi:10.1136/gutjnl-2011-300563
25. Annon JS (1976) The PLISSIT model: A proposed conceptual scheme for the behavioral treatment of sexual problems. Journal of Sex Education and Therapy 2 (1):1-15. doi:10.1080/01614576.1976.11074483
26. Basson R, Wierman ME, van Lankveld J, Brotto L (2010) Summary of the recommendations on sexual dysfunctions in women. The journal of sexual medicine 7 (1 Pt 2):314-326. doi:10.1111/j.1743-6109.2009.01617.x
27. Carter J, Goldfrank D, Schover LR (2011) Simple Strategies for Vaginal Health Promotion in Cancer Survivors. The journal of sexual medicine 8 (2):549-559. doi:10.1111/j.1743-6109.2010.01988.x
28. DuHamel K, Schuler T, Nelson C, Philip E, Temple L, Schover L, Baser RE, Starr TD, Cannon K, Jennings S, Jandorf L, Carter J (2016) The sexual health of female rectal and anal cancer survivors: results of a pilot randomized psycho-educational intervention trial. Journal of cancer survivorship : research and practice 10 (3):553-563. doi:10.1007/s11764-015-0501-8
29. Faithfull S, White I (2008) Delivering sensitive health care information: challenging the taboo of women's sexual health after pelvic radiotherapy. Patient education and counseling 71 (2):228-233. doi:10.1016/j.pec.2007.12.011
30. Bergmark K, Åvall-Lundqvist E, Dickman PW, Henningsohn L, Steineck G (2005) Synergy Between Sexual Abuse and Cervical Cancer in Causing Sexual Dysfunction. Journal of sex & marital therapy 31 (5):361-383. doi:10.1080/00926230591006476
31. Aoki Y, Brown HW, Brubaker L, Cornu JN, Daly JO, Cartwright R (2017) Urinary incontinence in women. Nature reviews Disease primers 3:17042-17042. doi:10.1038/nrdp.2017.42
32. Leddy LSJCUR (2018) Management of Lower Urinary Tract Symptoms After Pelvic Radiation in Females. 19 (12):106. doi:10.1007/s11934-018-0848-2
33. Lawenda BD, Mondry TE, Johnstone PAS (2009) Lymphedema: A primer on the identification and management of a chronic condition in oncologic treatment. 59 (1):8-24. doi:10.3322/caac.20001
34. Dunberger G, Lindquist H, Waldenström AC, Nyberg T, Steineck G, Åvall-Lundqvist E (2013) Lower limb lymphedema in gynecological cancer survivors--effect on daily life functioning. Supportive care

For peer review only

in cancer : official journal of the Multinational Association of Supportive Care in Cancer 21 (11):3063-3070. doi:10.1007/s00520-013-1879-3

35. Bakar Y, Tuğral A (2017) Lower Extremity Lymphedema Management after Gynecologic Cancer Surgery: A Review of Current Management Strategies. Annals of Vascular Surgery 44:442-450. doi:https://doi.org/10.1016/j.avsg.2017.03.197

36. Lindgren A, Dunberger G, Steineck G, Bergmark K, Enblom A (2020) Identifying female pelvic cancer survivors with low levels of physical activity after radiotherapy: women with fecal and urinary leakage need additional support. Supportive care in cancer : official journal of the Multinational Association of Supportive Care in Cancer 28 (6):2669-2681. doi:10.1007/s00520-019-05033-3

37. Åkeflo L, Elmerstig E, Dunberger G, Skokic V, Arnell A, Bergmark K (2021) Sexual health and wellbeing after pelvic radiotherapy among women with and without a reported history of sexual abuse: important issues in cancer survivorship care. Supportive care in cancer : official journal of the Multinational Association of Supportive Care in Cancer. doi:10.1007/s00520-021-06263-0

38. Schofield P, Juraskova I, Bergin R, Gough K, Mileshkin L, Krishnasamy M, White K, Bernshaw D, Penberthy S, Aranda S (2013) A nurse- and peer-led support program to assist women in gynaecological oncology receiving curative radiotherapy, the PeNTAGOn study (peer and nurse support trial to assist women in gynaecological oncology): study protocol for a randomised controlled trial. Trials 14:39. doi:10.1186/1745-6215-14-39

39. Jefford M, Aranda S, Gough K, Lotfi-Jam K, Butow P, Krishnasamy M, Young J, Phipps-Nelson J, Russell L, King D, Schofield P (2013) Evaluating a nurse-led survivorship care package (SurvivorCare) for bowel cancer survivors: study protocol for a randomized controlled trial. Trials 14:260-260. doi:10.1186/1745-6215-14-260

40. Hofsjo A, Bohm-Starke N, Blomgren B, Jahren H, Steineck G, Bergmark K (2017) Radiotherapy-induced vaginal fibrosis in cervical cancer survivors. Acta oncologica (Stockholm, Sweden):1-9. doi:10.1080/0284186x.2016.1275778

41. Westman B, Ullgren H, Olofsson A, Sharp L (2019) Patient-reported perceptions of care after the introduction of a new advanced cancer nursing role in Sweden. European Journal of Oncology Nursing 41:41-48. doi:10.1016/j.ejon.2019.05.009

42. Andersen B, Karlsson J, Anderson B, Tewfik H (1984) Anxiety and cancer treatment: Response to stressful radiotherapy. Health Psychology 3:535-551. doi:10.1037/0278-6133.3.6.535

43. Oberoi D, White VM, Seymour JF, Miles Prince H, Harrison S, Jefford M, Winship I, Hill D, Bolton D, Kay A, Millar J, Doo NW, Giles G (2017) The influence of unmet supportive care needs on anxiety and depression during cancer treatment and beyond: a longitudinal study of survivors of haematological cancers. Supportive care in cancer : official journal of the Multinational Association of Supportive Care in Cancer 25 (11):3447-3456. doi:10.1007/s00520-017-3766-9

44. Stubblefield MD (2017) The Underutilization of Rehabilitation to Treat Physical Impairments in Breast Cancer Survivors. 9 (9S2):S317-S323. doi:10.1016/j.pmrj.2017.05.010

45. Penalba V, Deshields TL, Klinkenberg DJSCiC (2019) Gaps in communication between cancer patients and healthcare providers: symptom distress and patients' intentions to disclose. 27 (6):2039-2047. doi:10.1007/s00520-018-4442-4

46. Rasmusson EM, Plantin L, Elmerstig E (2013) 'Did they think I would understand all that on my own?' A questionnaire study about sexuality with Swedish cancer patients. European journal of cancer care 22 (3):361-369. doi:10.1111/ecc.12039

47. Bakker RM, Mens JW, de Groot HE, Tuijnman-Raasveld CC, Braat C, Hompus WC, Poelman JG, Laman MS, Velema LA, de Kroon CD, van Doorn HC, Creutzberg CL, Ter Kuile MM (2017) A nurse-led sexual rehabilitation intervention after radiotherapy for gynecological cancer. Supportive care in cancer : official journal of the Multinational Association of Supportive Care in Cancer 25 (3):729-737. doi:10.1007/s00520-016-3453-2

48. WHO (2006) Sexual health and Sexuality, definition. http://www.who.int/reproductivehealth/topics/sexual_health/sh_definitions/en/.

For peer review only - http://bmjopen.bmj.com/site/about/guidelines.xhtml

49. Chapman CH, Heath G, Fairchild P, Berger MB, Wittmann D, Uppal S, Tolpadi A, Maturen K, Jolly SJJoCR, Oncology C (2019) Gynecologic radiation oncology patients report unmet needs regarding sexual health communication with providers. 145 (2):495-502. doi:10.1007/s00432-018-2813-3

50. Hordern AJ, Street AF (2007) Communicating about patient sexuality and intimacy after cancer: mismatched expectations and unmet needs. The Medical journal of Australia 186 (5):224-227

51. Concato J, Shah N, Horwitz RI (2000) Randomized, Controlled Trials, Observational Studies, and the Hierarchy of Research Designs. 342 (25):1887-1892. doi:10.1056/nejm200006223422507

52. Althubaiti A (2016) Information bias in health research: definition, pitfalls, and adjustment methods. J Multidiscip Healthc 9:211-217. doi:10.2147/JMDH.S104807

53. Steineck G, Bergmark K, Henningsohn L, al-Abany M, Dickman PW, Helgason A (2002) Symptom documentation in cancer survivors as a basis for therapy modifications. Acta oncologica (Stockholm, Sweden) 41 (3):244-252

54. Steineck G, Hunt H, Adolfsson J (2006) A hierarchical step-model for causation of bias-evaluating cancer treatment with epidemiological methods. Acta oncologica (Stockholm, Sweden) 45 (4):421-429. doi:10.1080/02841860600649293

For peer review only

Figure legends

Figure 1. *Flowchart of Pelvic Cancer Rehabilitation ongoing data collection procedure and the procedure for patient inclusion, contact with participants, and the follow-up points for questionnaires.*

Figure 2. *Schematic diagram of data collected from two different study cohorts from January 2011 to June 2017: a population-based cohort and patients referred from oncology healthcare providers, general practitioners, and through private referrals who met the inclusion criteria. The diagram includes study response rate, completeness of questionnaires, and reasons for loss of participants.*

For peer review only - http://bmjopen.bmj.com/site/about/guidelines.xhtml

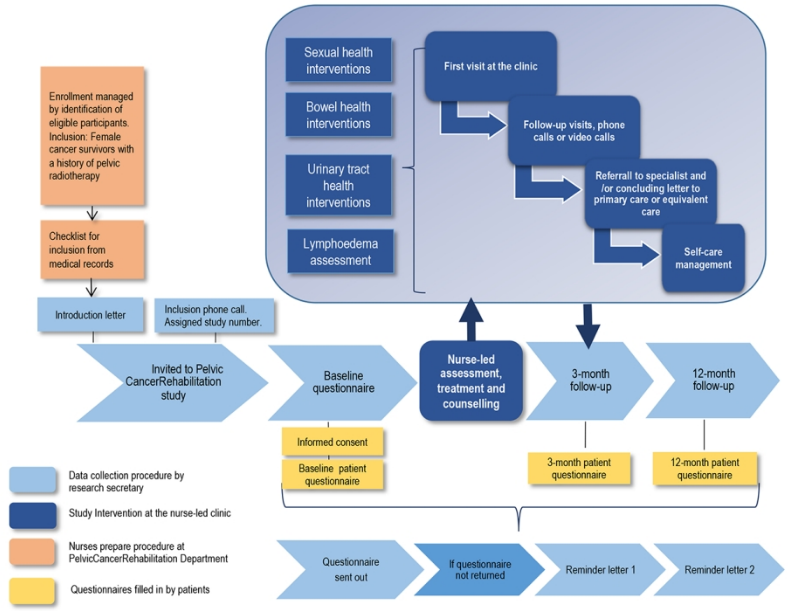

Figure 1. Flowchart of Pelvic Cancer Rehabilitation ongoing data collection procedure and the procedure for patient inclusion, contact with participants, and the follow-up points for questionnaires.

133x99mm (300 x 300 DPI)

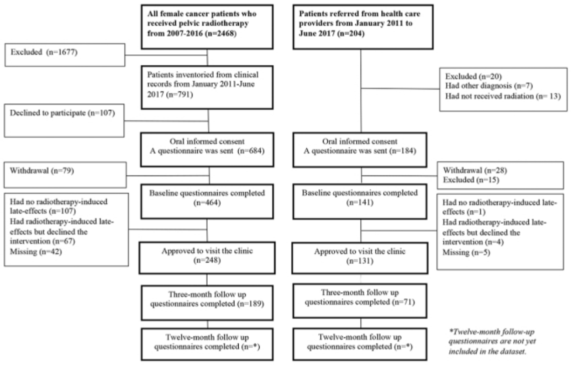

Figure 2. Schematic diagram of data collected from two different study cohorts from January 2011 to June 2017: a population-based cohort and patients referred from oncology healthcare providers, general practitioners, and through private referrals who met the inclusion criteria. The diagram includes study response rate, completeness of questionnaires, and reasons for loss of participants.

177x99mm (300 x 300 DPI)

STROBE (Strengthening The Reporting of OBservational Studies in Epidemiology) Checklist

A checklist of items that should be included in reports of observational studies. You must report the page number in your manuscript where you consider each of the items listed in this checklist. If you have not included this information, either revise your manuscript accordingly before submitting or note N/A.

Note: An Explanation and Elaboration article discusses each checklist item and gives methodological background and published examples of transparent reporting. The STROBE checklist is best used in conjunction with this article (freely available on the Web sites of PLoS Medicine at http://www.plosmedicine.org/, Annals of Internal Medicine at http://www.annals.org/, and Epidemiology at http://www.epidem.com/). Information on the STROBE Initiative is available at www.strobe-statement.org.

Section and Item	Item No.	Recommendation	Reported on Page No.
**Title and Abstract**	1	(*a*) Indicate the study's design with a commonly used term in the title or the abstract	
		(*b*) Provide in the abstract an informative and balanced summary of what was done and what was found	
**Introduction**			
Background/Rationale	2	Explain the scientific background and rationale for the investigation being reported	
Objectives	3	State specific objectives, including any prespecified hypotheses	
**Methods**			
Study Design	4	Present key elements of study design early in the paper	
Setting	5	Describe the setting, locations, and relevant dates, including periods of recruitment, exposure, follow-up, and data collection	
Participants	6	(*a*) *Cohort study*—Give the eligibility criteria, and the sources and methods of selection of participants. Describe methods of follow-up<br>*Case-control study*—Give the eligibility criteria, and the sources and methods of case ascertainment and control selection. Give the rationale for the choice of cases and controls<br>*Cross-sectional study*—Give the eligibility criteria, and the sources and methods of selection of participants	
		(*b*) *Cohort study*—For matched studies, give matching criteria and number of exposed and unexposed<br>*Case-control study*—For matched studies, give matching criteria and the number of controls per case	
Variables	7	Clearly define all outcomes, exposures, predictors, potential confounders, and effect modifiers. Give diagnostic criteria, if applicable	

For peer review only

Section and Item	Item No.	Recommendation	Reported on Page No.
Data Sources/ Measurement	8*	For each variable of interest, give sources of data and details of methods of assessment (measurement). Describe comparability of assessment methods if there is more than one group	
Bias	9	Describe any efforts to address potential sources of bias	
Study Size	10	Explain how the study size was arrived at	
Quantitative Variables	11	Explain how quantitative variables were handled in the analyses. If applicable, describe which groupings were chosen and why	
Statistical Methods	12	(*a*) Describe all statistical methods, including those used to control for confounding	
		(*b*) Describe any methods used to examine subgroups and interactions	
		(*c*) Explain how missing data were addressed	
		(*d*) *Cohort study*—If applicable, explain how loss to follow-up was addressed<br>*Case-control study*—If applicable, explain how matching of cases and controls was addressed<br>*Cross-sectional study*—If applicable, describe analytical methods taking account of sampling strategy	
		(*e*) Describe any sensitivity analyses	
**Results**			
Participants	13*	(a) Report numbers of individuals at each stage of study—eg numbers potentially eligible, examined for eligibility, confirmed eligible, included in the study, completing follow-up, and analysed	
		(b) Give reasons for non-participation at each stage	
		(c) Consider use of a flow diagram	
Descriptive Data	14*	(a) Give characteristics of study participants (eg demographic, clinical, social) and information on exposures and potential confounders	
		(b) Indicate number of participants with missing data for each variable of interest	
		(c) *Cohort study*—Summarise follow-up time (eg, average and total amount)	
Outcome Data	15*	*Cohort study*—Report numbers of outcome events or summary measures over time	
		Case-control study—Report numbers in each exposure category, or summary measures of exposure	
		Cross-sectional study—Report numbers of outcome events or summary measures	

For peer review only

Section and Item	Item No.	Recommendation	Reported on Page No.
Main Results	16	(*a*) Give unadjusted estimates and, if applicable, confounder-adjusted estimates and their precision (eg, 95% confidence interval). Make clear which confounders were adjusted for and why they were included	
		(*b*) Report category boundaries when continuous variables were categorized	
		(*c*) If relevant, consider translating estimates of relative risk into absolute risk for a meaningful time period	
Other Analyses	17	Report other analyses done—eg analyses of subgroups and interactions, and sensitivity analyses	
**Discussion**			
Key Results	18	Summarise key results with reference to study objectives	
Limitations	19	Discuss limitations of the study, taking into account sources of potential bias or imprecision. Discuss both direction and magnitude of any potential bias	
Interpretation	20	Give a cautious overall interpretation of results considering objectives, limitations, multiplicity of analyses, results from similar studies, and other relevant evidence	
Generalisability	21	Discuss the generalisability (external validity) of the study results	
**Other Information**			
Funding	22	Give the source of funding and the role of the funders for the present study and, if applicable, for the original study on which the present article is based	

*Give information separately for cases and controls in case-control studies and, if applicable, for exposed and unexposed groups in cohort and cross-sectional studies.

Once you have completed this checklist, please save a copy and upload it as part of your submission. DO NOT include this checklist as part of the main manuscript document. It must be uploaded as a separate file.

For peer review only
